# Supplementary material for: Hidden impacts of conservation management on fertility of the critically endangered kākāpō
Source: PeerJ. 2023 Feb 3;11:e14675. doi: 10.7717/peerj.14675 (PMC9901309; doi:10.7717/peerj.14675)
Supplement: Supplemental Information 1 — Information on each kākāpō clutch since 1990 and the number of kākāpō on each island, and R Markdown code and results to run the Bayesian model and calculate correlations between kākāpō abundance and frequency of multiple matings. [file peerj-11-14675-s001.zip › Supplemental_Data/Supplemental_Data_S3.html]

Kakapo Fertility Projpred modelling


Code 

- Show All Code
- Hide All Code
- Download Rmd

# Kakapo Fertility Projpred modelling

## Kakapo Fertility Projpred modelling

- 1 Credits
- 2 Purpose
- 3 Requirements
- 4 Data
- 5 Fertility model
  - 5.1 Load data
  - 5.2 Reference model
  - 5.3 Restricted variable selection
    - 5.3.1 Projection - full model
    - 5.3.2 Projection - reduced model
      - 5.3.2.1 Marginal means
- 6 Multiple copulation and kakapo density
  - 6.1 Number of copulations
  - 6.2 Number of kakapo
  - 6.3 Sex ratio
  - 6.4 Number and sex ratio combined
  - 6.5 Change over time
- 7 Session information

Alejandro Catalina and Andrew Digby

2023-01-18

```
set.seed(1111)
curr_dir <- setwd(dirname(rstudioapi::getSourceEditorContext()$path))
clutch_file <- file.path(curr_dir, "./Supplemental_Data_S1.csv")
kakapo_demog_file <- file.path(curr_dir, './Supplemental_Data_S2.csv')

outdir <- "./figure/"
ropeci <- 1.00 # proportion of posterior which intersects with ROPE. 
nchains <- 4
niter <- 30000 # iterations for brm
```

# 1 Credits

- Markdown from `readthedown` template, from package `rmdformats`.
- Code written by Alejandro Catalina and Andrew Digby.

# 2 Purpose

Code and results for the publication `Hidden impacts of conservation management on fertility of the critically endangered kakapo`. Model validation and diagnostics not included.

1. Use projected predictive variable selection package `projpred` to assess factors influencing kakapo fertility using a Bayesian model.
2. Compare multiple copulation frequency with kakapo density and sex ratio

# 3 Requirements

- Requires the `workflow` branch of `projpred`.

# 4 Data

Input data:

- Clutch fertility data frame from file Supplemental\_Data\_S1.csv.
- Kakapo numbers per island per year from Supplemental\_Data\_S2.csv.

# 5 Fertility model

## 5.1 Load data

```
ref_predfun <- function(fit, newdata = NULL) {
  return(t(posterior_linpred(fit,
                             newdata = newdata,
                             transform = FALSE
  )))
}

# Retrieve model data
extract_model_data <- function(object, newdata = NULL, wrhs = NULL,
                               orhs = NULL, extract_y = TRUE) {
  if (!extract_y) {
    resp_form <- NULL
  } else {
    resp_form <- ~.y
  }
  
  if (is.null(newdata)) {
    newdata <- data
  }
  
  if (is.null(wrhs) && !is.null(object) &&
      !is.null(object$weights) && length(object$weights) != 0) {
    wrhs <- ~weights
    newdata <- cbind(newdata, weights = object$weights)
  }
  
  if (is.null(orhs) && !is.null(object) &&
      !is.null(object$offset) && length(object$offset) != 0) {
    orhs <- ~offset
    newdata <- cbind(newdata, offset = object$offset)
  }
  
  args <- nlist(object, newdata, wrhs, orhs, resp_form)
  return(do_call(projpred:::.extract_model_data, args))
}

# For more readable parameter names in plots
fn_labels <- function(string){
  string <- sub('b_','',string)
  string <- gsub('scmother_age','Mother age',string)
  string <- gsub('scfather_age','Father age',string)
  string <- gsub('schandrear.mother','Mother HR',string)
  string <- gsub('schandrear.father','Father HR',string)
  string <- gsub('handrear.mother','Mother HR',string)
  string <- gsub('handrear.father','Father HR',string)
  string <- gsub('HRTRUE', 'HR', string)
  string <- gsub('scnumber.of.copulations','No. copulations',string)
  string <- gsub('scnumber.of.males','No. males',string)
  string <- gsub('sccopulations.mates','Mother copulations: ',string)
  string <- gsub('copulations.mates','Mother copulations: ',string)
  string <- gsub('Intercept','Intercept',string)
  string <- gsub('Differentmales','Different males',string)
  string <- gsub('2Pcopulations','1 male, >1 copulation',string)
  string <- gsub('2\\+ copulations','1 male, >1 copulation',string)
  string <- gsub('sckin','Mother/father kinship',string)
  string <- gsub("scprev.male.copulations", "Father previous copulations", string)
  string <- gsub("scprev.female.copulations", "Mother previous copulations", string)
  string <- gsub("sd_mother__Intercept", "SD(Mother intercept)", string)
  string <- gsub("sd_father__Intercept", "SD(Father intercept)", string)
  string <- gsub("sd_year__Intercept", "SD(Year intercept)", string)
  string <- gsub(": $","",string) # remove colon at end of string (to remove "Mating:" if no subsequent variable)
  
  return(string)

}
```

- Remove observations with missing values
- Scale and centre continuous predictors to mean of zero and SD=0.5 (see http://www.stat.columbia.edu/~gelman/research/published/priors11.pdf)

```
# Read clutch data file:
full_data <- read.csv(clutch_file, header = T, stringsAsFactors = T) %>%
  mutate(year = as.factor(year),
         handrear.mother = as.factor(handrear.mother),
         handrear.father = as.factor(handrear.father)) %>%
  rename_with(~ gsub("\\.","_", .x)) 
cat(sprintf("Loaded %d records from %s\n", nrow(full_data), basename(clutch_file)))
```

```
Loaded 225 records from Supplemental_Data_S1.csv
```

```
# Retain only variables of interest and drop rows with missing values
data <- full_data %>%  dplyr::select(clutch_fert, mother_age, handrear_mother, father_age,
         handrear_father, copulations_mates, prev_male_copulations, prev_female_copulations, kin, father, mother, year) %>%
  tidyr::drop_na()

# Scale and centre inputs to mean of zero & SD = 0.5
data <- data %>% mutate(
  scmother_age = 0.5*scale(mother_age, center=T, scale=T) ,
  scfather_age = 0.5*scale(father_age, center=T, scale=T),
  scprev_male_copulations = 0.5*scale(prev_male_copulations, center=T, scale=T),
  scprev_female_copulations = 0.5*scale(prev_female_copulations, center=T, scale=T),
  sckin = 0.5*scale(kin, center=T, scale=T),
)

# Convert matrix columns to vectors (result of using scale)
data <- data %>% mutate(scmother_age = as.vector(scmother_age),
                        scfather_age = as.vector(scfather_age),
                        sckin = as.vector(sckin),
                        scprev_male_copulations = as.vector(scprev_male_copulations),
                        scprev_female_copulations = as.vector(scprev_female_copulations))
```

Show predictor distributions for numeric variables

```
facetlabs <- c('mother_age'='Mother age (years)', 'father_age' = 'Father age (years)', 'prev_male_copulations' = 'Father previous copulations', 'prev_female_copulations' = 'Mother previous copulations', 'kin' = 'Mother/father kinship')
df <- data %>%  dplyr::select(mother_age, father_age, prev_male_copulations, prev_female_copulations, kin)
dfl <- df %>% pivot_longer(everything(), names_to='variable') %>% 
  mutate(variable = factor(variable, levels=c('mother_age', 'father_age', 'prev_male_copulations', 'prev_female_copulations', 'kin')))
fill_col1 <- brewer.pal(name='Set2',n=3)[1]
fill_col2 <- brewer.pal(name='Set2',n=3)[2]
fill_col3 <- brewer.pal(name='Set2',n=3)[3]
ggplot(dfl, aes(x=value)) + 
#  geom_histogram(data=subset(dfl, variable %in% c('mother_age', 'father_age', 'prev_male_copulations')), binwidth = 2, closed = 'left') +
  geom_histogram(data=subset(dfl, variable %in% c('mother_age', 'father_age')), breaks = seq(0, 50, 2), closed = 'right', fill = fill_col1, colour = 'black' ) +
  geom_histogram(data=subset(dfl, variable %in% c('prev_male_copulations')), breaks = seq(0,40,2), closed = 'right', fill = fill_col2, colour = 'black'  ) +
  geom_histogram(data=subset(dfl, variable %in% c('prev_female_copulations')), breaks = seq(0,20,1), closed = 'right', fill = fill_col2, colour = 'black'  ) +
  geom_histogram(data=subset(dfl, variable %in% c('kin')), breaks = seq(0, 0.3, 0.01), closed = 'right', fill = fill_col3, colour = 'black'  ) + 
  facet_wrap(~variable, scales='free', labeller=labeller(variable=facetlabs), ncol=2) +
    scale_x_continuous(expand = c(0.01,0)) +
  labs(y = 'Frequency', x = '') + 
  guides(fill = 'none')
```

```
 ggsave("./figure/figure_s2_predictor_distributions.pdf", height = 7, width = 8)
```

```
# Random variables:
grouping_variables <- c("father", "mother", "year")

# Predictors:
predictors <- c(
  "scmother_age", "handrear_mother", "scfather_age",
  "handrear_father", "copulations_mates", "scprev_male_copulations",
  "scprev_female_copulations", "sckin"
)
```

## 5.2 Reference model

Fit full reference model

```
# Formula for model
formula <- paste(
  "clutch_fert ~", paste0(predictors, collapse = " + "), " + ",
  paste(paste0("(1 | ", grouping_variables, ")"), collapse = " + "),
  "+ handrear_mother:handrear_father"
) %>%
  as.formula()
```

```
# Fit original model with {brms}
fit_original <- brm(formula,
  data = data, family = bernoulli(),
  prior = set_prior(horseshoe()),
  iter = niter, 
  chains = nchains,
  seed = 1111,
  control = list(adapt_delta = 0.99)
)
fit_original
```

```
 Family: bernoulli 
  Links: mu = logit 
Formula: clutch_fert ~ scmother_age + handrear_mother + scfather_age + handrear_father + copulations_mates + scprev_male_copulations + scprev_female_copulations + sckin + (1 | father) + (1 | mother) + (1 | year) + handrear_mother:handrear_father 
   Data: data (Number of observations: 217) 
  Draws: 4 chains, each with iter = 30000; warmup = 15000; thin = 1;
         total post-warmup draws = 60000

Group-Level Effects: 
~father (Number of levels: 50) 
              Estimate Est.Error l-95% CI u-95% CI Rhat Bulk_ESS Tail_ESS
sd(Intercept)     1.31      0.47     0.45     2.34 1.00    11905    11570

~mother (Number of levels: 60) 
              Estimate Est.Error l-95% CI u-95% CI Rhat Bulk_ESS Tail_ESS
sd(Intercept)     0.66      0.43     0.03     1.64 1.00     9430    17488

~year (Number of levels: 16) 
              Estimate Est.Error l-95% CI u-95% CI Rhat Bulk_ESS Tail_ESS
sd(Intercept)     0.65      0.51     0.03     1.91 1.00    13467    22681

Population-Level Effects: 
                                        Estimate Est.Error l-95% CI u-95% CI
Intercept                                   0.34      0.50    -0.67     1.33
scmother_age                               -0.11      0.37    -1.01     0.58
handrear_motherTRUE                        -0.18      0.42    -1.21     0.52
scfather_age                                0.25      0.46    -0.47     1.39
handrear_fatherTRUE                        -0.54      0.66    -2.10     0.36
copulations_mates2Pcopulations              0.50      0.53    -0.19     1.70
copulations_matesDifferentmales             1.04      0.60    -0.01     2.23
scprev_male_copulations                    -0.18      0.40    -1.20     0.46
scprev_female_copulations                  -0.24      0.41    -1.26     0.37
sckin                                      -0.21      0.34    -1.03     0.30
handrear_motherTRUE:handrear_fatherTRUE    -0.25      0.58    -1.69     0.73
                                        Rhat Bulk_ESS Tail_ESS
Intercept                               1.00    26170    34085
scmother_age                            1.00    54597    52449
handrear_motherTRUE                     1.00    47172    54689
scfather_age                            1.00    41212    47335
handrear_fatherTRUE                     1.00    29749    48986
copulations_mates2Pcopulations          1.00    24999    48199
copulations_matesDifferentmales         1.00    22274    14814
scprev_male_copulations                 1.00    35644    47661
scprev_female_copulations               1.00    40404    51617
sckin                                   1.00    33317    45872
handrear_motherTRUE:handrear_fatherTRUE 1.00    51141    53152

Draws were sampled using sampling(NUTS). For each parameter, Bulk_ESS
and Tail_ESS are effective sample size measures, and Rhat is the potential
scale reduction factor on split chains (at convergence, Rhat = 1).
```

## 5.3 Restricted variable selection

```
# Set up variable selection
## run variable selection on the unconstrained latent space
ref_original <- brms:::get_refmodel.brmsfit(fit_original, seed = 1111)
f_latent <- colMeans(posterior_linpred(fit_original, transform = FALSE))
data[[".y"]] <- f_latent
dis_latent <- rep(1, 2*niter)

# Get reference model
ref <- init_refmodel(fit_original,
 data = data, formula = formula, family = gaussian(),
 ref_predfun = ref_predfun, div_minimizer = projpred:::linear_multilevel_mle,
 proj_predfun = projpred:::linear_multilevel_proj_predfun, dis = dis_latent,
 extract_model_data = extract_model_data, cvfun = ref_original$cvfun,
 seed = 1111
)
```

Constrain the variable selection to select the fixed terms first, then the random terms.

```
# Order variables for selection: first fixed terms, then random terms
search_terms <- c(
  "1",
  "handrear_mother + handrear_father + handrear_mother:handrear_father",
  "scmother_age",
  "handrear_mother",
  "scfather_age",
  "handrear_father",
  "copulations_mates",
  "scprev_male_copulations",
  "scprev_female_copulations",
  "sckin",
  paste(
    "scmother_age + handrear_mother + scfather_age + handrear_father +",
    "copulations_mates + scprev_male_copulations + scprev_female_copulations + sckin +",
    "handrear_mother:handrear_father + (1 | father)"
  ),
  paste(
    "scmother_age + handrear_mother + scfather_age + handrear_father +",
    "copulations_mates + scprev_male_copulations + scprev_female_copulations + sckin +",
    "handrear_mother:handrear_father + (1 | mother)"
  ),
  paste(
    "scmother_age + handrear_mother + scfather_age + handrear_father +",
    "copulations_mates + scprev_male_copulations + scprev_female_copulations + sckin +",
    "handrear_mother:handrear_father + (1 | year)"
  )
)
```

```
vs_restricted <- varsel(ref, search_terms = search_terms, seed=1111)
```

```
[1] "10% of terms selected."
[1] "20% of terms selected."
[1] "30% of terms selected."
[1] "40% of terms selected."
[1] "50% of terms selected."
[1] "60% of terms selected."
[1] "70% of terms selected."
[1] "80% of terms selected."
[1] "90% of terms selected."
[1] "100% of terms selected."
```

```
summary(vs_restricted, stats = c('elpd'), type = c('mean', 'se', 'lower', 'upper', 'diff', 'diff_se'), deltas = T)
```

```
Family: gaussian 
Link function: identity 

Formula: clutch_fert ~ scmother_age + handrear_mother + scfather_age + 
    handrear_father + copulations_mates + scprev_male_copulations + 
    scprev_female_copulations + sckin + (1 | father) + (1 | mother) + 
    (1 | year) + handrear_mother:handrear_father
<environment: 0x10e7435f0>
Observations: 217
Search method: forward, maximum number of terms 12
Draws used for selection: 20, in 20 clusters
Draws used for prediction: 400
Suggested Projection Size: 11

Selection Summary:
 size                  solution_terms   elpd  se  lower upper
    0                            <NA> -101.2 3.4 -104.6 -97.8
    1                 handrear_father  -79.3 2.9  -82.2 -76.4
    2               copulations_mates  -65.6 2.3  -67.9 -63.3
    3                 handrear_mother  -61.4 2.4  -63.8 -59.0
    4                    scfather_age  -59.6 2.5  -62.0 -57.1
    5       scprev_female_copulations  -57.7 2.6  -60.3 -55.1
    6 handrear_father:handrear_mother  -57.4 2.6  -60.0 -54.9
    7                    scmother_age  -57.3 2.5  -59.8 -54.8
    8         scprev_male_copulations  -57.2 2.5  -59.7 -54.7
    9                           sckin  -57.1 2.5  -59.7 -54.6
   10                    (1 | father)   -5.3 0.5   -5.9  -4.8
   11                    (1 | mother)   -0.1 0.4   -0.5   0.3
   12                      (1 | year)    0.0 0.3   -0.3   0.3
```

```
vs_binom <- vs_restricted

# Project onto latent space and then back to binomial: get better results than if just projecting original full model.
ll_fun <- ref_original$family$ll_fun
linkinv <- ref_original$family$linkinv
for (k in seq_along(vs_restricted$summaries$sub)) {
  vs_binom$summaries$sub[[k]]$mu <- linkinv(vs_restricted$summaries$sub[[k]]$mu)
  vs_binom$summaries$sub[[k]]$draws <-
    linkinv(vs_restricted$summaries$sub[[k]]$draws)
  lppd <- ll_fun(
    mu=vs_binom$summaries$sub[[k]]$draws,
    dis=NULL, y=as.numeric(as.matrix(data[, "clutch_fert"])),
    weights = ref$wobs
  )
  vs_binom$summaries$sub[[k]]$lppd <- apply(
    lppd, 1, projpred:::log_weighted_mean_exp, rep(1 / NCOL(lppd), NCOL(lppd))
  )
}

# Transform back to binomial space:
vs_binom$summaries$ref$mu <- linkinv(vs_restricted$summaries$ref$mu)
vs_binom$summaries$ref$draws <- linkinv(vs_restricted$summaries$ref$draws)
lppd <- ll_fun(
  vs_binom$summaries$ref$draws,
  NULL, as.numeric(as.matrix(data[, "clutch_fert"])),
  weights = ref$wobs
)
vs_binom$summaries$ref$lppd <- apply(
  lppd, 1, projpred:::log_weighted_mean_exp, ref$wsample
)
```

```
## Plot variable selection results
elpd_replace <- function(string){
  string <- paste(toupper(string), " difference vs reference model")
}
plot(vs_binom, stats = c('elpd'), deltas=T, alpha=0.32) +
  scale_x_continuous(breaks = c(0, seq_along(vs_restricted$solution_terms)),
                     labels = c("Intercept", fn_labels(vs_restricted$solution_terms))) +
  scale_y_continuous(expand = c(0,0)) +
  expand_limits(y=c(-60,1)) +
  facet_wrap(statistic ~ ., strip.position = "left", labeller = labeller(statistic=elpd_replace)) +
  theme(axis.text.x = element_text(angle = 30, hjust = 1, vjust = 1),
        axis.ticks = element_line(),
        panel.grid.major.x = element_blank(),
        panel.grid.minor.x = element_blank(),
        panel.grid.major.y = element_blank(),
        panel.grid.minor.y = element_blank(),
        strip.text = element_blank(), # no facet text
        strip.placement = "outside",
        strip.background = element_blank(),
      panel.border = element_rect(fill = NA, colour = 'black')) +
  labs(x='Terms in the submodel', y = 'ELPD difference from the reference model')
```

```
ggsave(file.path(outdir,"figure_1_varsel.pdf"), height = 6, width = 8)
```

```
# Makek table of variablel selection results
d <- summary(vs_binom, type=c('mean', 'se', 'diff', 'lower', 'upper'), stats=c('elpd'), deltas=T)

# Add contribution of difference to ELPD for each term, from the previous term:
tot_elpd <- d$selection$elpd[nrow(d$selection)] - d$selection$elpd[1]
dsel <- d$selection %>% mutate(elpd_diff = elpd - lag(elpd),
                       elpd_diff_pc = 100 * elpd_diff / tot_elpd)
format(dsel, digits=2)
```

```
   size                  solution_terms   elpd   se  lower upper elpd_diff
1     0                              NA -52.00 4.97 -56.95 -47.1        NA
2     1                 handrear_father -44.45 5.13 -49.55 -39.3    7.5529
3     2               copulations_mates -38.98 4.77 -43.73 -34.2    5.4651
4     3                 handrear_mother -37.76 4.60 -42.33 -33.2    1.2217
5     4                    scfather_age -37.52 4.54 -42.03 -33.0    0.2401
6     5       scprev_female_copulations -36.42 4.49 -40.88 -32.0    1.1045
7     6 handrear_father:handrear_mother -36.41 4.50 -40.89 -31.9    0.0035
8     7                    scmother_age -36.37 4.45 -40.80 -31.9    0.0427
9     8         scprev_male_copulations -36.35 4.41 -40.74 -32.0    0.0193
10    9                           sckin -36.20 4.40 -40.58 -31.8    0.1503
11   10                    (1 | father) -10.46 1.56 -12.01  -8.9   25.7451
12   11                    (1 | mother)  -4.30 0.92  -5.22  -3.4    6.1542
13   12                      (1 | year)  -0.16 0.27  -0.43   0.1    4.1392
   elpd_diff_pc
1            NA
2       14.5701
3       10.5425
4        2.3568
5        0.4631
6        2.1307
7        0.0068
8        0.0823
9        0.0373
10       0.2900
11      49.6639
12      11.8718
13       7.9848
```

```
# Random term contributions to ELPD:
cat(sprintf("Contribution of random model terms to ELPD difference = %.1f%%", dsel %>% filter(grepl("|", solution_terms, fixed=T)) %>% summarise(tot_elpd_diff_pc = sum(elpd_diff_pc)) %>% as.numeric()))
```

```
Contribution of random model terms to ELPD difference = 69.5%
```

### 5.3.1 Projection - full model

Projected marginals of the full model

```
# Set the solution terms - all terms
if (suggest_size(vs_binom) > length(vs_binom$solution_terms)){
  solution_terms <- vs_binom$solution_terms[seq_len(suggest_size(vs_binom)-1)]
} else {
  solution_terms <- vs_binom$solution_terms[seq_len(suggest_size(vs_binom))]
}
projection <- projpred::project(vs_restricted, solution_terms = solution_terms, seed=1111)

#Project onto latent space and then back to binomial: get better results than if just projecting original full model.
ll_fun <- ref_original$family$ll_fun
linkinv <- ref_original$family$linkinv
for (k in seq_along(projection$summaries$sub)) {
  projection$summaries$sub[[k]]$mu <- linkinv(projection$summaries$sub[[k]]$mu)
  projection$summaries$sub[[k]]$draws <-
    linkinv(projection$summaries$sub[[k]]$draws)
  lppd <- ll_fun(
    projection$summaries$sub[[k]]$draws,
    NULL, as.numeric(as.matrix(data[, "clutch_fert"])),
    weights = ref$wobs
  )
  projection$summaries$sub[[k]]$lppd <- apply(
    lppd, 1, projpred:::log_weighted_mean_exp, rep(1 / NCOL(lppd), NCOL(lppd))
  )
}

# Project back to binomial space
projection$summaries$ref$mu <- linkinv(projection$summaries$ref$mu)
projection$summaries$ref$draws <- vs_binom$summaries$ref$draws
lppd <- ll_fun(
  projection$summaries$ref$draws,
  NULL, as.numeric(as.matrix(data[, "clutch_fert"])),
  weights = ref$wobs
)
projection$summaries$ref$lppd <- apply(
  lppd, 1, projpred:::log_weighted_mean_exp, ref$wsample
)
```

R^2 for original model:

```
# Compare with brms::bayes_R2 function
bayes_R2(fit_original)
```

```
    Estimate  Est.Error      Q2.5     Q97.5
R2 0.3124318 0.07100593 0.1709913 0.4513358
```

R^2 for full projection:

```
# Modified function to calculate R2 for projected model
# Modified from https://avehtari.github.io/bayes_R2/bayes_R2.html#1_Introduction
bayes_R2_res <- function(proj) {
  y <- get_y(proj$refmodel$fit)
  # Use proj_linpred instead of rstanarm::posterior_epred
  #   use $pred to get values in response space
  ypred_latent <- proj_linpred(proj, transform = T)$pred
  # Apply inverse link function to output of proj_linpred to convert from latent function
  ypred <- ref_original$family$linkinv(ypred_latent)

  if (proj$refmodel$fit$family$family == "binomial" && NCOL(y) == 2) {
    trials <- rowSums(y)
    y <- y[, 1]
    ypred <- ypred %*% diag(trials)
  }
  e <- -1 * sweep(ypred, 2, y)
  var_ypred <- apply(ypred, 1, var)
  var_e <- apply(e, 1, var)
  r2 <- var_ypred / (var_ypred + var_e)
    r2 %>% as.data.frame(.) %>%
    summarise(Mean = mean(r2), SD = sd(r2), Q2.5 = quantile(r2, 0.025), Q97.5 = quantile(r2, 0.975))

}
(R2_res <- bayes_R2_res(projection))
```

```
       Mean         SD      Q2.5     Q97.5
1 0.3114775 0.07144011 0.1651088 0.4516667
```

ROPE plot and posterior statistics. Use `rope_range` to specify range of ROPE, which is -0.1813799, 0.1813799 for logistic regression.

```
# Plot ROPE
mp <- as.data.frame(as.matrix(projection)) %>% 
  select(!starts_with("r_"), -sigma) # don't plot random factors or sigma

# Add median and HDI
gmp <- pivot_longer(mp %>% select(-b_Intercept), cols=everything(), names_to='variable')

# Retain order of variables for plot:
gmp$variable <- fct_relevel(gmp$variable, names(mp))
gmp$y <- gmp$variable
(p <- plot(rope(mp,ci=ropeci, range=rope_range(fit_original))) + 
    stat_pointinterval(data=gmp, aes(y=factor(variable, levels=names(mp)), x=value, height=NULL,fill=NULL), point_interval=median_hdi, .width=c(0.5, 0.95), normalize='xy',point_size=3, point_colour='black', interval_colour='blue', shape=21, point_fill='yellow', alpha=0.5) +
    scale_y_discrete(labels=fn_labels, expand = expansion(add = c(-0,1.2)))  +
    scale_x_continuous(labels = function(x)x/2) + # to convert posterior scale from 0.5 SD to SD
    labs(title='', y = 'Variable', x = "Possible values (SD)")  +
    guides(fill = 'none') 
)
```

```
ggsave(file.path(outdir, "figure_2_posterior_full.pdf"), height = 6, width = 8)

# Posterior statistics
names(mp) <- fn_labels(names(mp))
describe_posterior(mp, ci_method='hdi', ci=0.95, rope_ci=ropeci, rope_range=rope_range(fit_original), centrality = "median",test = c("p_direction", "p_significance", "rope", 'equivalence_test'))
```

```
Summary of Posterior Distribution

Parameter                                 | Median |        95% CI |     pd |   ps |          ROPE | % in ROPE | Equivalence (ROPE)
-----------------------------------------------------------------------------------------------------------------------------------
Intercept                                 |   0.43 | [-0.44, 1.25] | 85.25% | 0.73 | [-0.18, 0.18] |    19.25% |          Undecided
Father HR                                 |  -0.76 | [-2.00, 0.19] | 94.00% | 0.87 | [-0.18, 0.18] |    10.75% |          Undecided
Mother copulations: 1 male, >1 copulation |   0.35 | [-0.26, 1.64] | 81.75% | 0.61 | [-0.18, 0.18] |    35.25% |          Undecided
Mother copulations: Different males       |   1.11 | [-0.03, 2.05] | 96.25% | 0.90 | [-0.18, 0.18] |     9.50% |          Undecided
Mother HR                                 |  -0.16 | [-1.24, 0.56] | 71.00% | 0.48 | [-0.18, 0.18] |    39.00% |          Undecided
Father age                                |   0.11 | [-0.62, 1.16] | 64.75% | 0.43 | [-0.18, 0.18] |    43.75% |          Undecided
Mother previous copulations               |  -0.12 | [-1.06, 0.48] | 72.50% | 0.43 | [-0.18, 0.18] |    50.00% |          Undecided
Mother age                                |  -0.06 | [-0.98, 0.45] | 64.75% | 0.34 | [-0.18, 0.18] |    51.25% |          Undecided
Father previous copulations               |  -0.04 | [-1.00, 0.58] | 63.00% | 0.34 | [-0.18, 0.18] |    55.00% |          Undecided
Mother/father kinship                     |  -0.12 | [-1.01, 0.31] | 79.25% | 0.45 | [-0.18, 0.18] |    50.00% |          Undecided
Father HR:Mother HR                       |  -0.06 | [-1.48, 0.75] | 64.00% | 0.36 | [-0.18, 0.18] |    51.75% |          Undecided
SD(Mother intercept)                      |   0.53 | [ 0.00, 1.17] |   100% | 0.85 | [-0.18, 0.18] |    14.75% |          Undecided
SD(Father intercept)                      |   0.85 | [ 0.32, 1.67] |   100% | 0.99 | [-0.18, 0.18] |     1.00% |           Rejected
SD(Year intercept)                        |   0.41 | [ 0.00, 1.00] |   100% | 0.83 | [-0.18, 0.18] |    17.25% |          Undecided
```

### 5.3.2 Projection - reduced model

Project only the `copulations` and `hand-rearing` fixed predictors, since these contribute the most to the variance and have non-zero posteriors in the full model, plus the random terms

```
# Set solution terms: best two fixed terms and all random terms
solution_terms <- c(vs_binom$solution_terms[1:2],
                     vs_binom$solution_terms[10:12])
  projection <- projpred::project(vs_restricted, solution_terms = solution_terms, seed=1111)

# Project onto latent space and then back to binomial: get better results than if just projecting original full model.
ll_fun <- ref_original$family$ll_fun
linkinv <- ref_original$family$linkinv
for (k in seq_along(projection$summaries$sub)) {
  projection$summaries$sub[[k]]$mu <- linkinv(projection$summaries$sub[[k]]$mu)
  projection$summaries$sub[[k]]$draws <-
    linkinv(projection$summaries$sub[[k]]$draws)
  lppd <- ll_fun(
    projection$summaries$sub[[k]]$draws,
    NULL, as.numeric(as.matrix(data[, "clutch_fert"])),
    weights = ref$wobs
  )
  projection$summaries$sub[[k]]$lppd <- apply(
    lppd, 1, projpred:::log_weighted_mean_exp, rep(1 / NCOL(lppd), NCOL(lppd))
  )
}

# Project back to binomial
projection$summaries$ref$mu <- linkinv(projection$summaries$ref$mu)
projection$summaries$ref$draws <- vs_binom$summaries$ref$draws
lppd <- ll_fun(
  projection$summaries$ref$draws,
  NULL, as.numeric(as.matrix(data[, "clutch_fert"])),
  weights = ref$wobs
)
projection$summaries$ref$lppd <- apply(
  lppd, 1, projpred:::log_weighted_mean_exp, ref$wsample
)
```

R2 of projected reduced model:

```
(R2_res <- bayes_R2_res(projection))
```

```
       Mean         SD      Q2.5     Q97.5
1 0.3065322 0.07057051 0.1618977 0.4478909
```

ROPE plot and posterior statistics:

```
# Plot ROPE
mp <- as.data.frame(as.matrix(projection))%>% 
  select(!starts_with("r_"), -sigma) # don't plot random factors or sigma

# Add median and HDI
gmp <- pivot_longer(mp %>% select(-b_Intercept), cols=everything(), names_to='variable')

# Retain order of variables:
gmp$variable <- fct_relevel(gmp$variable, names(mp))
gmp$y <- gmp$variable
plot(rope(mp,ci=ropeci, range=rope_range(fit_original))) + 
  stat_pointinterval(data=gmp, aes(y=factor(variable, levels=names(mp)), x=value, height=NULL,fill=NULL), point_interval=median_hdi, .width=c(0.5, 0.95), normalize='xy',point_size=3, point_colour='black', interval_colour='blue', shape=21,point_fill='yellow', alpha=0.5) +
  scale_y_discrete(labels=fn_labels, expand = expansion(add = c(-0,1.2))) + 
      scale_x_continuous(labels = function(x)x/2) + # to convert posterior scale from 0.5 SD to SD
    labs(title='', y = 'Variable', x = "Possible values (SD)")  +
  guides(fill='none')
```

```
ggsave(file.path(outdir, "figure_3_posterior_reduced.pdf"), height = 6, width = 8)

# Posterior stats:
names(mp) <- fn_labels(names(mp))
describe_posterior(mp, ci_method='hdi', ci=0.95, rope_ci=ropeci, rope_range=rope_range(fit_original), centrality = "median",test = c("p_direction", "p_significance", "rope", 'equivalence_test'))
```

```
Summary of Posterior Distribution

Parameter                                 | Median |        95% CI |     pd |   ps |          ROPE | % in ROPE | Equivalence (ROPE)
-----------------------------------------------------------------------------------------------------------------------------------
Intercept                                 |   0.58 | [-0.22, 1.51] | 92.25% | 0.84 | [-0.18, 0.18] |    13.50% |          Undecided
Father HR                                 |  -1.03 | [-2.08, 0.16] | 97.50% | 0.93 | [-0.18, 0.18] |     6.50% |          Undecided
Mother copulations: 1 male, >1 copulation |   0.33 | [-0.24, 1.56] | 81.00% | 0.60 | [-0.18, 0.18] |    35.75% |          Undecided
Mother copulations: Different males       |   1.14 | [-0.03, 2.06] | 96.75% | 0.92 | [-0.18, 0.18] |     8.50% |          Undecided
SD(Mother intercept)                      |   0.65 | [ 0.05, 1.36] |   100% | 0.95 | [-0.18, 0.18] |     5.25% |          Undecided
SD(Father intercept)                      |   1.27 | [ 0.30, 2.18] |   100% | 1.00 | [-0.18, 0.18] |     0.25% |           Rejected
SD(Year intercept)                        |   0.63 | [ 0.00, 1.61] | 98.75% | 0.92 | [-0.18, 0.18] |     8.25% |          Undecided
```

#### 5.3.2.1 Marginal means

Evaluate interaction plot of estimated marginal means from the projected posterior for hand-rearing and number of copulations combined:

```
# Create projection dataframe
mpb <- as.data.frame(as.matrix(projection)) %>% select(starts_with("b_")) %>% rename_with(.fn=function(x) gsub("b_","",x), .cols=everything())  %>% as.matrix(.)

# Grid for copulations_mates
grd_hr2 <- qdrg(~ copulations_mates + handrear_father , data=fit_original$data, mcmc=mpb[, c('Intercept','copulations_mates2+ copulations', 'copulations_matesDifferent males', 'handrear_fatherTRUE')], link='logit')


# Plot without data:
proj.int <- emmip(grd_hr2, formula("~copulations_mates  + handrear_father"), type='response', plotit=F) %>%
  mutate(father_rear= case_when(handrear_father=='TRUE' ~ 'Hand-reared',
                                 handrear_father=='FALSE' ~ 'Wild-reared'))
ghr_mate <- ggplot() + 
  geom_pointrange(data=proj.int, aes(x=copulations_mates, y=yvar, ymin=LCL, ymax=UCL, colour=father_rear, shape = father_rear), position=position_dodge(width=0.1))  + 
  labs(y='Probability of clutch fertility', x='', colour='Father rearing', shape = 'Father rearing') + 
  scale_x_discrete(labels=fn_labels) +
  scale_colour_brewer(palette = 'Set2', direction = -1) # colour-blind friendly

ggsave(file.path(outdir, "figure_4_predict.pdf"), ghr_mate, height = 6, width = 8)


# Plot with data:
ghr_mate + 
  geom_jitter(data=fit_original$data %>% 
                mutate(father_rear= case_when(
                  handrear_father=='TRUE' ~ 'Hand-reared',
                  handrear_father=='FALSE' ~ 'Wild-reared')), 
              aes(x=copulations_mates, y=clutch_fert, colour=father_rear, shape = father_rear), alpha=0.5, width=0.1, height=0.05)
```

```
ggsave(file.path(outdir, "figure_4_predict_data.pdf"), height = 6, width = 8)

# Results table:
proj.int %>% rename(clutch.fertility = yvar) %>%
  mutate(father_rear= case_when(handrear_father=='TRUE' ~ 'Hand-reared',
                                 handrear_father=='FALSE' ~ 'Wild-reared')) %>%
  select(copulations_mates, father_rear, clutch.fertility, LCL, UCL) %>%
  format(., digits=3)
```

```
  copulations_mates father_rear clutch.fertility   LCL   UCL
1      1 copulation Wild-reared            0.641 0.468 0.837
2    2+ copulations Wild-reared            0.718 0.524 0.924
3   Different males Wild-reared            0.842 0.666 0.972
4      1 copulation Hand-reared            0.385 0.154 0.732
5    2+ copulations Hand-reared            0.504 0.202 0.833
6   Different males Hand-reared            0.658 0.348 0.932
```

# 6 Multiple copulation and kakapo density

```
# Read number of kakapo per island per year
nk <- read.csv(kakapo_demog_file, header = T) 

# Number of copulations/males per island per year per number of matings/mates
tc <- table(full_data$Island, full_data$year, full_data$copulations_mates)
dtc <- data.frame(tc) %>% rename(Island = Var1, year = Var2, copulations = Var3) %>% mutate(year = as.numeric(as.character(year)))

# Total clutches per island:
dtc_tot <- dtc %>% group_by(year, Island) %>% summarise(totclutch=sum(Freq))
dtc <- left_join(dtc, dtc_tot, by=c('year', 'Island'))

# Combine dataframes: number of kakapo per island and copulations proportions
nk2 <- left_join(dtc, nk %>% filter(ageClass=='Adult'), by=c('year', 'Island'), suffix = c('.cop', '.kak')) %>%
  mutate(prop.kak = Freq.cop / Freq.kak,
         prop.clutch = Freq.cop / totclutch) %>%
  filter(!is.na(Sex) & !is.na(Freq.kak) & Freq.kak > 0)
```

Combine repeated copulations with the same male and copulations with different males to compare single vs multiple copulations.

Only consider Whenua Hou from 1990 onwards.

```
nk2 <- nk2 %>% mutate(copulations_comb = fct_collapse(copulations, 
                                              single = "1 copulation",
                                              multiple = c("2+ copulations", "Different males")))
# Totals:
nkg <- nk2 %>% 
  group_by(Island, year, Sex, copulations_comb) %>% 
  summarise(Freq.cop=sum(Freq.cop, na.rm=T), Freq.kak = mean(Freq.kak, na.rm = T), totclutch = mean(totclutch, na.rm = T)) %>%
  mutate(prop.clutch = Freq.cop / totclutch)

# Only Whenua Hou from 1990:
nkgsub <- nkg %>% filter(Island=='Whenua Hou' & year>=1990 & copulations_comb == 'multiple')
```

## 6.1 Number of copulations

Show table number of copulations vs number of mates

```
table(`Number of mates` = full_data$number_of_males, `Number of copulations` = full_data$number_of_copulations)
```

```
               Number of copulations
Number of mates   1   2   3   4
              1 105  46   4   0
              2   0  45  14   2
              3   0   0   1   1
```

## 6.2 Number of kakapo

Multiple copulation proportion vs number of males and females

```
# Calculate correlation:
(cor <- nkgsub %>% ungroup(.) %>% dplyr::select(Sex, Freq.kak, prop.clutch) %>% group_by( Sex) %>% correlation())
```

```
# Correlation Matrix (pearson-method)

Group  | Parameter1 |  Parameter2 |    r |        95% CI | t(8) |         p
---------------------------------------------------------------------------
Female |   Freq.kak | prop.clutch | 0.93 | [ 0.74, 0.98] | 7.44 | < .001***
Male   |   Freq.kak | prop.clutch | 0.61 | [-0.02, 0.90] | 2.20 | 0.059    

p-value adjustment method: Holm (1979)
Observations: 10
```

```
cor$p
```

```
[1] 7.319028e-05 5.875909e-02
```

```
# Base plot:
col <- brewer.pal(name='Set1',n=3)[2]
gwh1990_comb <- ggplot(nkgsub %>% filter(!is.na(prop.clutch)), aes(x= Freq.kak, y = prop.clutch)) + 
  geom_point(size=2, colour=col) + 
  geom_smooth(aes(shape=NULL), fill=col, method=lm, alpha=0.2) + 
  labs(y = "Proportion of clutches with multiple copulations", x = 'Number of adult kakapo')  + 
  facet_wrap(~Sex, scales='free_x')
```

## 6.3 Sex ratio

Multiple copulation proportion vs sex ratio:

```
# Calculate sex ratio
srg <- nkgsub %>% 
  pivot_wider(id_cols = c(Island, year, copulations_comb, Freq.cop, totclutch, prop.clutch), names_from = 'Sex', values_from="Freq.kak") %>%
  mutate(sex.ratio = Female/Male)

# Calculation correlation:
(corsex <- srg %>% ungroup(.) %>% dplyr::select(sex.ratio, prop.clutch)  %>% correlation(bayesian=F))
```

```
# Correlation Matrix (pearson-method)

Parameter1 |  Parameter2 |    r |       95% CI | t(8) |         p
-----------------------------------------------------------------
sex.ratio  | prop.clutch | 0.92 | [0.71, 0.98] | 6.88 | < .001***

p-value adjustment method: Holm (1979)
Observations: 10
```

```
corsex$p
```

```
[1] 0.0001274216
```

```
col <- brewer.pal(name='Set1',n=3)[1]
# Plot of multiple copulation frequency vs sex ratio
gswh1990comb <- ggplot(srg %>% filter(copulations_comb=='multiple'), aes(x= sex.ratio, y=prop.clutch)) + 
  geom_point(size=2, colour=col) + geom_smooth(aes(shape=NULL), method=lm, alpha=0.2, colour=col, fill=col) + 
  labs(y = "Prop. of clutches with multiple copulations", x = 'Female:male sex ratio')
```

## 6.4 Number and sex ratio combined

Combine number of male/female and sex ratio plots:

```
# Separate panels for females, males and sex ratio
# Multiple copulation frequency vs number of females
col <- brewer.pal(name='Set2',n=3)[1] # colour-blind friendly
gwh1990_female <- ggplot(nkgsub %>% filter(!is.na(prop.clutch) & Sex == 'Female'), aes(x= Freq.kak, y = prop.clutch)) + 
  geom_point(size=2, colour=col) + 
  geom_smooth(aes(shape=NULL), colour = col, fill=col, method=lm, alpha=0.2) + 
  labs(y = "Proportion of clutches", x = 'Number of females') +
  coord_cartesian(ylim = c(0,1)) + 
  expand_limits(x=40)

# Multiple copulation frequency vs number of males
col <- brewer.pal(name='Set2',n=3)[2]
gwh1990_male <- ggplot(nkgsub %>% filter(!is.na(prop.clutch) & Sex == 'Male'), aes(x= Freq.kak, y = prop.clutch)) + 
  geom_point(size=2, colour=col) + 
  geom_smooth(aes(shape=NULL), fill=col, method=lm, alpha=0.2, colour=col, fill=col) + 
  labs(y = "", x = 'Number of males')  +
  coord_cartesian(ylim = c(0,1)) + 
  expand_limits(x=c(15,30))

# Multiple copulation frequency vs sex ratio
col <- brewer.pal(name='Set2',n=3)[3]
gwh1990_sexratio <- ggplot(srg %>% filter(copulations_comb=='multiple'), aes(x= sex.ratio, y=prop.clutch)) + 
  geom_point(size=2, colour=col) + 
  geom_smooth(aes(shape=NULL), method=lm, alpha=0.2, colour=col, fill=col) + 
  labs(y = "", x = 'Female:male sex ratio') +
  coord_cartesian(ylim = c(0,1)) + 
  expand_limits(x=0.4) 

ggarrange(gwh1990_female,
          gwh1990_male,
          gwh1990_sexratio,
          common.legend = T , 
          labels = c("A", "B", "C"),
          label.x = 0.25,
          label.y = 0.98,
          ncol = 3,
          nrow = 1,
          widths=c(1,1,1))
```

```
# For publication:
ggsave(filename = file.path(outdir, 'figure_5_copulation_sex_ratio.pdf'), plot=last_plot(), device = 'pdf', width=10, height=6 )
```

## 6.5 Change over time

Show the change in Whenua Hou population and sex ratio over time (note that these data include some years in which there was no breeding):

```
# Total population
ggplot(srg %>% filter(!is.na(sex.ratio))  , aes(x = year, y = Female + Male)) +
         geom_point() + 
         geom_line() +
    scale_x_continuous(breaks = seq(min(srg$year), max(srg$year), 5), minor_breaks = c(min(srg$year): max(srg$year))) + 
  scale_y_continuous(breaks = seq(0,1,0.2)) +
  labs(y = 'Number of adult kakapo')
```

```
# Male and female population
ggplot(srg %>% filter(!is.na(sex.ratio) & !is.nan(prop.clutch)) %>% pivot_longer(cols = Female:Male, names_to = 'variable') , aes(x = year, y = value, colour = variable)) +
         geom_point() + 
         geom_line() +
  facet_wrap(~variable, scales = 'fixed') + 
  guides(colour = 'none') + 
    scale_x_continuous(breaks = seq(min(srg$year), max(srg$year), 5), minor_breaks = c(min(srg$year): max(srg$year))) + 
  labs(y = 'Number of adult kakapo')
```

```
# Sex ratio
ggplot(srg %>% filter(!is.na(sex.ratio)& !is.nan(prop.clutch)) , aes(x = year, y = sex.ratio)) +
         geom_point() + 
         geom_line() +
    scale_x_continuous(breaks = seq(min(srg$year), max(srg$year), 5), minor_breaks = c(min(srg$year): max(srg$year))) + 
  labs(y = 'F:M adult sex ratio')
```

Change in multiple copulation rate over time:

```
# Sex ratio
ggplot(srg %>% filter(!is.na(sex.ratio)& !is.nan(prop.clutch)) , aes(x = year, y = prop.clutch)) +
         geom_point() + 
         geom_line() +
  scale_x_continuous(breaks = seq(min(srg$year), max(srg$year), 5), minor_breaks = c(min(srg$year): max(srg$year))) + 
  scale_y_continuous(breaks = seq(0,1,0.2)) +
  labs(y = 'Proportion of multiple copulations')
```

# 7 Session information

```
sessionInfo()
```

```
R version 4.1.2 (2021-11-01)
Platform: aarch64-apple-darwin20 (64-bit)
Running under: macOS Monterey 12.6.2

Matrix products: default
BLAS:   /Library/Frameworks/R.framework/Versions/4.1-arm64/Resources/lib/libRblas.0.dylib
LAPACK: /Library/Frameworks/R.framework/Versions/4.1-arm64/Resources/lib/libRlapack.dylib

locale:
[1] en_US.UTF-8/en_US.UTF-8/en_US.UTF-8/C/en_US.UTF-8/en_US.UTF-8

attached base packages:
[1] stats     graphics  grDevices utils     datasets  methods   base     

other attached packages:
 [1] ggpubr_0.4.0         RColorBrewer_1.1-3   correlation_0.8.0   
 [4] emmeans_1.7.3        loo_2.5.1            brms_2.16.3         
 [7] Rcpp_1.0.9           doRNG_1.8.2          rngtools_1.5.2      
[10] doFuture_0.12.1      future_1.24.0        foreach_1.5.2       
[13] ggdist_3.1.1         see_0.6.9            bayestestR_0.11.5   
[16] bayesplot_1.9.0      lme4_1.1-29          Matrix_1.4-1        
[19] rstan_2.21.5         StanHeaders_2.21.0-7 forcats_0.5.1       
[22] stringr_1.4.0        dplyr_1.0.9          purrr_0.3.4         
[25] readr_2.1.1          tidyr_1.1.4          tibble_3.1.7        
[28] ggplot2_3.3.6        tidyverse_1.3.2      projpred_2.0.5.9000 

loaded via a namespace (and not attached):
  [1] readxl_1.3.1         backports_1.4.1      plyr_1.8.7          
  [4] igraph_1.2.11        splines_4.1.2        crosstalk_1.2.0     
  [7] listenv_0.8.0        optimx_2021-10.12    rstantools_2.2.0    
 [10] inline_0.3.19        digest_0.6.29        htmltools_0.5.2     
 [13] fansi_1.0.3          magrittr_2.0.3       checkmate_2.1.0     
 [16] googlesheets4_1.0.0  tzdb_0.2.0           globals_0.14.0      
 [19] modelr_0.1.8         RcppParallel_5.1.5   matrixStats_0.62.0  
 [22] xts_0.12.1           rmdformats_1.0.3     prettyunits_1.1.1   
 [25] colorspace_2.0-3     rvest_1.0.2          haven_2.4.3         
 [28] xfun_0.28            callr_3.7.0          crayon_1.5.1        
 [31] jsonlite_1.8.0       zoo_1.8-9            iterators_1.0.14    
 [34] glue_1.6.2           gtable_0.3.0         gargle_1.2.0        
 [37] distributional_0.3.0 car_3.0-12           pkgbuild_1.3.0      
 [40] abind_1.4-5          scales_1.2.0         mvtnorm_1.1-3       
 [43] DBI_1.1.1            rstatix_0.7.0        miniUI_0.1.1.1      
 [46] xtable_1.8-4         HDInterval_0.2.2     DT_0.20             
 [49] stats4_4.1.2         datawizard_0.4.0     htmlwidgets_1.5.4   
 [52] httr_1.4.2           threejs_0.3.3        posterior_1.2.1     
 [55] ellipsis_0.3.2       pkgconfig_2.0.3      farver_2.1.0        
 [58] sass_0.4.0           dbplyr_2.1.1         utf8_1.2.2          
 [61] labeling_0.4.2       reshape2_1.4.4       tidyselect_1.1.2    
 [64] rlang_1.0.2          later_1.3.0          munsell_0.5.0       
 [67] cellranger_1.1.0     tools_4.1.2          cli_3.3.0           
 [70] generics_0.1.2       broom_0.7.10         ggridges_0.5.3      
 [73] evaluate_0.15        fastmap_1.1.0        yaml_2.3.5          
 [76] processx_3.5.3       knitr_1.36           fs_1.5.2            
 [79] nlme_3.1-153         mime_0.12            xml2_1.3.3          
 [82] shinythemes_1.2.0    compiler_4.1.2       rstudioapi_0.13     
 [85] gamm4_0.2-6          ggsignif_0.6.3       reprex_2.0.1        
 [88] bslib_0.3.1          stringi_1.7.6        parameters_0.17.0   
 [91] highr_0.9            ps_1.7.0             Brobdingnag_1.2-8   
 [94] lattice_0.20-45      markdown_1.1         nloptr_2.0.0        
 [97] shinyjs_2.1.0        tensorA_0.36.2       vctrs_0.4.1         
[100] pillar_1.7.0         lifecycle_1.0.1      jquerylib_0.1.4     
[103] bridgesampling_1.1-2 estimability_1.3     cowplot_1.1.1       
[106] insight_0.17.0       httpuv_1.6.5         R6_2.5.1            
[109] bookdown_0.24        promises_1.2.0.1     gridExtra_2.3       
[112] parallelly_1.30.0    codetools_0.2-18     gtools_3.9.2        
[115] colourpicker_1.1.1   boot_1.3-28          MASS_7.3-54         
[118] assertthat_0.2.1     withr_2.5.0          shinystan_2.6.0     
[121] mgcv_1.8-38          parallel_4.1.2       hms_1.1.1           
[124] grid_4.1.2           coda_0.19-4          minqa_1.2.4         
[127] rmarkdown_2.11       carData_3.0-5        googledrive_2.0.0   
[130] numDeriv_2016.8-1.1  shiny_1.7.1          lubridate_1.8.0     
[133] base64enc_0.1-3      dygraphs_1.1.1.6
```

LS0tCnRpdGxlOiAiS2FrYXBvIEZlcnRpbGl0eSBQcm9qcHJlZCBtb2RlbGxpbmciCmF1dGhvcjogIkFsZWphbmRybyBDYXRhbGluYSBhbmQgQW5kcmV3IERpZ2J5IgpkYXRlOiAiYHIgU3lzLkRhdGUoKWAiCm91dHB1dDogCiAgcm1kZm9ybWF0czo6cmVhZHRoZWRvd246CiAgICAgbGlnaHRib3g6IHRydWUKICAgICB0b2NfZGVwdGg6IDUKICAgICBnYWxsZXJ5OiB0cnVlCiAgICAgZmlnX2NhcHRpb246IHRydWUKICAgICBjb2RlX2ZvbGRpbmc6IGhpZGUKICAgICBjb2RlX2Rvd25sb2FkOiB0cnVlCiAgICAgdXNlX2Jvb2tkb3duOiB0cnVlCmVkaXRvcl9vcHRpb25zOiAKICBjaHVua19vdXRwdXRfdHlwZTogaW5saW5lCi0tLQoKYGBge3Igc2V0dXAsIGVjaG89RkFMU0UsIG1lc3NhZ2U9RkFMU0UsIGluY2x1ZGU9RkFMU0V9CmtuaXRyOjpvcHRzX2NodW5rJHNldChkZXY9YygncG5nJywncGRmJyksIGNvbW1lbnQ9IiIsIGZpZy53aWR0aD0xMCwgZWNobz1UUlVFLCB3YXJuaW5nPUZBTFNFLCBjYWNoZT1GLCBtZXNzYWdlPUZBTFNFLCBmaWcucGF0aD0nZmlndXJlLycpCgojIFJlcXVpcmVzIHdvcmtmbG93IGJyYW5jaCBvZiBwcm9qcHJlZDoKI2xpYnJhcnkoZGV2dG9vbHMpCiNpbnN0YWxsX2dpdGh1YihyZXBvID0gInN0YW4tZGV2L3Byb2pwcmVkIiwgcmVmID0gIndvcmtmbG93IikKCmxpYnJhcnkocHJvanByZWQpCmxpYnJhcnkodGlkeXZlcnNlKSAKbGlicmFyeShyc3RhbikKbGlicmFyeShsbWU0KQpsaWJyYXJ5KGJheWVzcGxvdCkKbGlicmFyeShiYXllc3Rlc3RSKQpsaWJyYXJ5KHNlZSkKbGlicmFyeShnZ2Rpc3QpCmxpYnJhcnkoZG9GdXR1cmUpCmxpYnJhcnkoZG9STkcpCmxpYnJhcnkoYnJtcykKbGlicmFyeShsb28pCmxpYnJhcnkoZW1tZWFucykKbGlicmFyeShjb3JyZWxhdGlvbikKbGlicmFyeShSQ29sb3JCcmV3ZXIpCmxpYnJhcnkoZ2dwdWJyKQoKcnN0YW5fb3B0aW9ucyAoYXV0b193cml0ZT1UUlVFKQpvcHRpb25zIChtYy5jb3Jlcz1wYXJhbGxlbDo6ZGV0ZWN0Q29yZXMgKCkpICMgUnVuIG9uIG11bHRpcGxlIGNvcmVzCgp0aGVtZV9zZXQodGhlbWVfbWluaW1hbChiYXNlX3NpemUgPSAxNCkgKyB0aGVtZSgKICAgICAgICBheGlzLnRpY2tzID0gZWxlbWVudF9saW5lKCksCiAgICAgIHBhbmVsLmdyaWQubWFqb3IueCA9IGVsZW1lbnRfYmxhbmsoKSwKICAgICAgcGFuZWwuZ3JpZC5taW5vci54ID0gZWxlbWVudF9ibGFuaygpLAogICAgICBwYW5lbC5ncmlkLm1pbm9yLnkgPSBlbGVtZW50X2JsYW5rKCksCiAgICAgIHBhbmVsLmdyaWQubWFqb3IueSA9IGVsZW1lbnRfYmxhbmsoKSwKICAgICAgcGFuZWwuYm9yZGVyID0gZWxlbWVudF9yZWN0KGZpbGwgPSBOQSwgY29sb3VyID0gJ2JsYWNrJykpKQpgYGAKCmBgYHtyIHBhcmFtZXRlcnN9CnNldC5zZWVkKDExMTEpCmN1cnJfZGlyIDwtIHNldHdkKGRpcm5hbWUocnN0dWRpb2FwaTo6Z2V0U291cmNlRWRpdG9yQ29udGV4dCgpJHBhdGgpKQpjbHV0Y2hfZmlsZSA8LSBmaWxlLnBhdGgoY3Vycl9kaXIsICIuL1N1cHBsZW1lbnRhbF9EYXRhX1MxLmNzdiIpCmtha2Fwb19kZW1vZ19maWxlIDwtIGZpbGUucGF0aChjdXJyX2RpciwgJy4vU3VwcGxlbWVudGFsX0RhdGFfUzIuY3N2JykKCm91dGRpciA8LSAiLi9maWd1cmUvIgpyb3BlY2kgPC0gMS4wMCAjIHByb3BvcnRpb24gb2YgcG9zdGVyaW9yIHdoaWNoIGludGVyc2VjdHMgd2l0aCBST1BFLiAKbmNoYWlucyA8LSA0Cm5pdGVyIDwtIDMwMDAwICMgaXRlcmF0aW9ucyBmb3IgYnJtCmBgYAoKIyBDcmVkaXRzCgotICAgTWFya2Rvd24gZnJvbSBgcmVhZHRoZWRvd25gIHRlbXBsYXRlLCBmcm9tIHBhY2thZ2UgYHJtZGZvcm1hdHNgLgotICAgQ29kZSB3cml0dGVuIGJ5IEFsZWphbmRybyBDYXRhbGluYSBhbmQgQW5kcmV3IERpZ2J5LgoKIyBQdXJwb3NlCgpDb2RlIGFuZCByZXN1bHRzIGZvciB0aGUgcHVibGljYXRpb24gYEhpZGRlbiBpbXBhY3RzIG9mIGNvbnNlcnZhdGlvbiBtYW5hZ2VtZW50IG9uIGZlcnRpbGl0eSBvZiB0aGUgY3JpdGljYWxseSBlbmRhbmdlcmVkIGtha2Fwb2AuIE1vZGVsIHZhbGlkYXRpb24gYW5kIGRpYWdub3N0aWNzIG5vdCBpbmNsdWRlZC4KCjEuICBVc2UgcHJvamVjdGVkIHByZWRpY3RpdmUgdmFyaWFibGUgc2VsZWN0aW9uIHBhY2thZ2UgYHByb2pwcmVkYCB0byBhc3Nlc3MgZmFjdG9ycyBpbmZsdWVuY2luZyBrYWthcG8gZmVydGlsaXR5IHVzaW5nIGEgQmF5ZXNpYW4gbW9kZWwuCjIuICBDb21wYXJlIG11bHRpcGxlIGNvcHVsYXRpb24gZnJlcXVlbmN5IHdpdGgga2FrYXBvIGRlbnNpdHkgYW5kIHNleCByYXRpbwoKIyBSZXF1aXJlbWVudHMKCiAtIFJlcXVpcmVzIHRoZSBgd29ya2Zsb3dgIGJyYW5jaCBvZiBgcHJvanByZWRgLgogCiMgRGF0YQoKSW5wdXQgZGF0YToKCi0gICBDbHV0Y2ggZmVydGlsaXR5IGRhdGEgZnJhbWUgZnJvbSBmaWxlIGByIGJhc2VuYW1lKGNsdXRjaF9maWxlKWAuCi0gICBLYWthcG8gbnVtYmVycyBwZXIgaXNsYW5kIHBlciB5ZWFyIGZyb20gYHIgYmFzZW5hbWUoa2FrYXBvX2RlbW9nX2ZpbGUpYC4KCiMgRmVydGlsaXR5IG1vZGVsCgojIyBMb2FkIGRhdGEKCmBgYHtyIGZ1bmN0aW9uc30KCnJlZl9wcmVkZnVuIDwtIGZ1bmN0aW9uKGZpdCwgbmV3ZGF0YSA9IE5VTEwpIHsKICByZXR1cm4odChwb3N0ZXJpb3JfbGlucHJlZChmaXQsCiAgICAgICAgICAgICAgICAgICAgICAgICAgICAgbmV3ZGF0YSA9IG5ld2RhdGEsCiAgICAgICAgICAgICAgICAgICAgICAgICAgICAgdHJhbnNmb3JtID0gRkFMU0UKICApKSkKfQoKIyBSZXRyaWV2ZSBtb2RlbCBkYXRhCmV4dHJhY3RfbW9kZWxfZGF0YSA8LSBmdW5jdGlvbihvYmplY3QsIG5ld2RhdGEgPSBOVUxMLCB3cmhzID0gTlVMTCwKICAgICAgICAgICAgICAgICAgICAgICAgICAgICAgIG9yaHMgPSBOVUxMLCBleHRyYWN0X3kgPSBUUlVFKSB7CiAgaWYgKCFleHRyYWN0X3kpIHsKICAgIHJlc3BfZm9ybSA8LSBOVUxMCiAgfSBlbHNlIHsKICAgIHJlc3BfZm9ybSA8LSB+LnkKICB9CiAgCiAgaWYgKGlzLm51bGwobmV3ZGF0YSkpIHsKICAgIG5ld2RhdGEgPC0gZGF0YQogIH0KICAKICBpZiAoaXMubnVsbCh3cmhzKSAmJiAhaXMubnVsbChvYmplY3QpICYmCiAgICAgICFpcy5udWxsKG9iamVjdCR3ZWlnaHRzKSAmJiBsZW5ndGgob2JqZWN0JHdlaWdodHMpICE9IDApIHsKICAgIHdyaHMgPC0gfndlaWdodHMKICAgIG5ld2RhdGEgPC0gY2JpbmQobmV3ZGF0YSwgd2VpZ2h0cyA9IG9iamVjdCR3ZWlnaHRzKQogIH0KICAKICBpZiAoaXMubnVsbChvcmhzKSAmJiAhaXMubnVsbChvYmplY3QpICYmCiAgICAgICFpcy5udWxsKG9iamVjdCRvZmZzZXQpICYmIGxlbmd0aChvYmplY3Qkb2Zmc2V0KSAhPSAwKSB7CiAgICBvcmhzIDwtIH5vZmZzZXQKICAgIG5ld2RhdGEgPC0gY2JpbmQobmV3ZGF0YSwgb2Zmc2V0ID0gb2JqZWN0JG9mZnNldCkKICB9CiAgCiAgYXJncyA8LSBubGlzdChvYmplY3QsIG5ld2RhdGEsIHdyaHMsIG9yaHMsIHJlc3BfZm9ybSkKICByZXR1cm4oZG9fY2FsbChwcm9qcHJlZDo6Oi5leHRyYWN0X21vZGVsX2RhdGEsIGFyZ3MpKQp9CgojIEZvciBtb3JlIHJlYWRhYmxlIHBhcmFtZXRlciBuYW1lcyBpbiBwbG90cwpmbl9sYWJlbHMgPC0gZnVuY3Rpb24oc3RyaW5nKXsKICBzdHJpbmcgPC0gc3ViKCdiXycsJycsc3RyaW5nKQogIHN0cmluZyA8LSBnc3ViKCdzY21vdGhlcl9hZ2UnLCdNb3RoZXIgYWdlJyxzdHJpbmcpCiAgc3RyaW5nIDwtIGdzdWIoJ3NjZmF0aGVyX2FnZScsJ0ZhdGhlciBhZ2UnLHN0cmluZykKICBzdHJpbmcgPC0gZ3N1Yignc2NoYW5kcmVhci5tb3RoZXInLCdNb3RoZXIgSFInLHN0cmluZykKICBzdHJpbmcgPC0gZ3N1Yignc2NoYW5kcmVhci5mYXRoZXInLCdGYXRoZXIgSFInLHN0cmluZykKICBzdHJpbmcgPC0gZ3N1YignaGFuZHJlYXIubW90aGVyJywnTW90aGVyIEhSJyxzdHJpbmcpCiAgc3RyaW5nIDwtIGdzdWIoJ2hhbmRyZWFyLmZhdGhlcicsJ0ZhdGhlciBIUicsc3RyaW5nKQogIHN0cmluZyA8LSBnc3ViKCdIUlRSVUUnLCAnSFInLCBzdHJpbmcpCiAgc3RyaW5nIDwtIGdzdWIoJ3NjbnVtYmVyLm9mLmNvcHVsYXRpb25zJywnTm8uIGNvcHVsYXRpb25zJyxzdHJpbmcpCiAgc3RyaW5nIDwtIGdzdWIoJ3NjbnVtYmVyLm9mLm1hbGVzJywnTm8uIG1hbGVzJyxzdHJpbmcpCiAgc3RyaW5nIDwtIGdzdWIoJ3NjY29wdWxhdGlvbnMubWF0ZXMnLCdNb3RoZXIgY29wdWxhdGlvbnM6ICcsc3RyaW5nKQogIHN0cmluZyA8LSBnc3ViKCdjb3B1bGF0aW9ucy5tYXRlcycsJ01vdGhlciBjb3B1bGF0aW9uczogJyxzdHJpbmcpCiAgc3RyaW5nIDwtIGdzdWIoJ0ludGVyY2VwdCcsJ0ludGVyY2VwdCcsc3RyaW5nKQogIHN0cmluZyA8LSBnc3ViKCdEaWZmZXJlbnRtYWxlcycsJ0RpZmZlcmVudCBtYWxlcycsc3RyaW5nKQogIHN0cmluZyA8LSBnc3ViKCcyUGNvcHVsYXRpb25zJywnMSBtYWxlLCA+MSBjb3B1bGF0aW9uJyxzdHJpbmcpCiAgc3RyaW5nIDwtIGdzdWIoJzJcXCsgY29wdWxhdGlvbnMnLCcxIG1hbGUsID4xIGNvcHVsYXRpb24nLHN0cmluZykKICBzdHJpbmcgPC0gZ3N1Yignc2NraW4nLCdNb3RoZXIvZmF0aGVyIGtpbnNoaXAnLHN0cmluZykKICBzdHJpbmcgPC0gZ3N1Yigic2NwcmV2Lm1hbGUuY29wdWxhdGlvbnMiLCAiRmF0aGVyIHByZXZpb3VzIGNvcHVsYXRpb25zIiwgc3RyaW5nKQogIHN0cmluZyA8LSBnc3ViKCJzY3ByZXYuZmVtYWxlLmNvcHVsYXRpb25zIiwgIk1vdGhlciBwcmV2aW91cyBjb3B1bGF0aW9ucyIsIHN0cmluZykKICBzdHJpbmcgPC0gZ3N1Yigic2RfbW90aGVyX19JbnRlcmNlcHQiLCAiU0QoTW90aGVyIGludGVyY2VwdCkiLCBzdHJpbmcpCiAgc3RyaW5nIDwtIGdzdWIoInNkX2ZhdGhlcl9fSW50ZXJjZXB0IiwgIlNEKEZhdGhlciBpbnRlcmNlcHQpIiwgc3RyaW5nKQogIHN0cmluZyA8LSBnc3ViKCJzZF95ZWFyX19JbnRlcmNlcHQiLCAiU0QoWWVhciBpbnRlcmNlcHQpIiwgc3RyaW5nKQogIHN0cmluZyA8LSBnc3ViKCI6ICQiLCIiLHN0cmluZykgIyByZW1vdmUgY29sb24gYXQgZW5kIG9mIHN0cmluZyAodG8gcmVtb3ZlICJNYXRpbmc6IiBpZiBubyBzdWJzZXF1ZW50IHZhcmlhYmxlKQogIAogIHJldHVybihzdHJpbmcpCgp9CmBgYAoKLSAgIFJlbW92ZSBvYnNlcnZhdGlvbnMgd2l0aCBtaXNzaW5nIHZhbHVlcwotICAgU2NhbGUgYW5kIGNlbnRyZSBjb250aW51b3VzIHByZWRpY3RvcnMgdG8gbWVhbiBvZiB6ZXJvIGFuZCBTRD0wLjUgKHNlZSBodHRwOi8vd3d3LnN0YXQuY29sdW1iaWEuZWR1L35nZWxtYW4vcmVzZWFyY2gvcHVibGlzaGVkL3ByaW9yczExLnBkZikKCmBgYHtyIHJlYWRfZGF0YX0KIyBSZWFkIGNsdXRjaCBkYXRhIGZpbGU6CmZ1bGxfZGF0YSA8LSByZWFkLmNzdihjbHV0Y2hfZmlsZSwgaGVhZGVyID0gVCwgc3RyaW5nc0FzRmFjdG9ycyA9IFQpICU+JQogIG11dGF0ZSh5ZWFyID0gYXMuZmFjdG9yKHllYXIpLAogICAgICAgICBoYW5kcmVhci5tb3RoZXIgPSBhcy5mYWN0b3IoaGFuZHJlYXIubW90aGVyKSwKICAgICAgICAgaGFuZHJlYXIuZmF0aGVyID0gYXMuZmFjdG9yKGhhbmRyZWFyLmZhdGhlcikpICU+JQogIHJlbmFtZV93aXRoKH4gZ3N1YigiXFwuIiwiXyIsIC54KSkgCmNhdChzcHJpbnRmKCJMb2FkZWQgJWQgcmVjb3JkcyBmcm9tICVzXG4iLCBucm93KGZ1bGxfZGF0YSksIGJhc2VuYW1lKGNsdXRjaF9maWxlKSkpICAKCiMgUmV0YWluIG9ubHkgdmFyaWFibGVzIG9mIGludGVyZXN0IGFuZCBkcm9wIHJvd3Mgd2l0aCBtaXNzaW5nIHZhbHVlcwpkYXRhIDwtIGZ1bGxfZGF0YSAlPiUgIGRwbHlyOjpzZWxlY3QoY2x1dGNoX2ZlcnQsIG1vdGhlcl9hZ2UsIGhhbmRyZWFyX21vdGhlciwgZmF0aGVyX2FnZSwKICAgICAgICAgaGFuZHJlYXJfZmF0aGVyLCBjb3B1bGF0aW9uc19tYXRlcywgcHJldl9tYWxlX2NvcHVsYXRpb25zLCBwcmV2X2ZlbWFsZV9jb3B1bGF0aW9ucywga2luLCBmYXRoZXIsIG1vdGhlciwgeWVhcikgJT4lCiAgdGlkeXI6OmRyb3BfbmEoKQoKIyBTY2FsZSBhbmQgY2VudHJlIGlucHV0cyB0byBtZWFuIG9mIHplcm8gJiBTRCA9IDAuNQpkYXRhIDwtIGRhdGEgJT4lIG11dGF0ZSgKICBzY21vdGhlcl9hZ2UgPSAwLjUqc2NhbGUobW90aGVyX2FnZSwgY2VudGVyPVQsIHNjYWxlPVQpICwKICBzY2ZhdGhlcl9hZ2UgPSAwLjUqc2NhbGUoZmF0aGVyX2FnZSwgY2VudGVyPVQsIHNjYWxlPVQpLAogIHNjcHJldl9tYWxlX2NvcHVsYXRpb25zID0gMC41KnNjYWxlKHByZXZfbWFsZV9jb3B1bGF0aW9ucywgY2VudGVyPVQsIHNjYWxlPVQpLAogIHNjcHJldl9mZW1hbGVfY29wdWxhdGlvbnMgPSAwLjUqc2NhbGUocHJldl9mZW1hbGVfY29wdWxhdGlvbnMsIGNlbnRlcj1ULCBzY2FsZT1UKSwKICBzY2tpbiA9IDAuNSpzY2FsZShraW4sIGNlbnRlcj1ULCBzY2FsZT1UKSwKKQoKIyBDb252ZXJ0IG1hdHJpeCBjb2x1bW5zIHRvIHZlY3RvcnMgKHJlc3VsdCBvZiB1c2luZyBzY2FsZSkKZGF0YSA8LSBkYXRhICU+JSBtdXRhdGUoc2Ntb3RoZXJfYWdlID0gYXMudmVjdG9yKHNjbW90aGVyX2FnZSksCiAgICAgICAgICAgICAgICAgICAgICAgIHNjZmF0aGVyX2FnZSA9IGFzLnZlY3RvcihzY2ZhdGhlcl9hZ2UpLAogICAgICAgICAgICAgICAgICAgICAgICBzY2tpbiA9IGFzLnZlY3RvcihzY2tpbiksCiAgICAgICAgICAgICAgICAgICAgICAgIHNjcHJldl9tYWxlX2NvcHVsYXRpb25zID0gYXMudmVjdG9yKHNjcHJldl9tYWxlX2NvcHVsYXRpb25zKSwKICAgICAgICAgICAgICAgICAgICAgICAgc2NwcmV2X2ZlbWFsZV9jb3B1bGF0aW9ucyA9IGFzLnZlY3RvcihzY3ByZXZfZmVtYWxlX2NvcHVsYXRpb25zKSkKCmBgYAoKClNob3cgcHJlZGljdG9yIGRpc3RyaWJ1dGlvbnMgZm9yIG51bWVyaWMgdmFyaWFibGVzCgpgYGB7ciBtb2RkYXRhX2Rpc3RyaWJ9CmZhY2V0bGFicyA8LSBjKCdtb3RoZXJfYWdlJz0nTW90aGVyIGFnZSAoeWVhcnMpJywgJ2ZhdGhlcl9hZ2UnID0gJ0ZhdGhlciBhZ2UgKHllYXJzKScsICdwcmV2X21hbGVfY29wdWxhdGlvbnMnID0gJ0ZhdGhlciBwcmV2aW91cyBjb3B1bGF0aW9ucycsICdwcmV2X2ZlbWFsZV9jb3B1bGF0aW9ucycgPSAnTW90aGVyIHByZXZpb3VzIGNvcHVsYXRpb25zJywgJ2tpbicgPSAnTW90aGVyL2ZhdGhlciBraW5zaGlwJykKZGYgPC0gZGF0YSAlPiUgIGRwbHlyOjpzZWxlY3QobW90aGVyX2FnZSwgZmF0aGVyX2FnZSwgcHJldl9tYWxlX2NvcHVsYXRpb25zLCBwcmV2X2ZlbWFsZV9jb3B1bGF0aW9ucywga2luKQpkZmwgPC0gZGYgJT4lIHBpdm90X2xvbmdlcihldmVyeXRoaW5nKCksIG5hbWVzX3RvPSd2YXJpYWJsZScpICU+JSAKICBtdXRhdGUodmFyaWFibGUgPSBmYWN0b3IodmFyaWFibGUsIGxldmVscz1jKCdtb3RoZXJfYWdlJywgJ2ZhdGhlcl9hZ2UnLCAncHJldl9tYWxlX2NvcHVsYXRpb25zJywgJ3ByZXZfZmVtYWxlX2NvcHVsYXRpb25zJywgJ2tpbicpKSkKZmlsbF9jb2wxIDwtIGJyZXdlci5wYWwobmFtZT0nU2V0Micsbj0zKVsxXQpmaWxsX2NvbDIgPC0gYnJld2VyLnBhbChuYW1lPSdTZXQyJyxuPTMpWzJdCmZpbGxfY29sMyA8LSBicmV3ZXIucGFsKG5hbWU9J1NldDInLG49MylbM10KZ2dwbG90KGRmbCwgYWVzKHg9dmFsdWUpKSArIAojICBnZW9tX2hpc3RvZ3JhbShkYXRhPXN1YnNldChkZmwsIHZhcmlhYmxlICVpbiUgYygnbW90aGVyX2FnZScsICdmYXRoZXJfYWdlJywgJ3ByZXZfbWFsZV9jb3B1bGF0aW9ucycpKSwgYmlud2lkdGggPSAyLCBjbG9zZWQgPSAnbGVmdCcpICsKICBnZW9tX2hpc3RvZ3JhbShkYXRhPXN1YnNldChkZmwsIHZhcmlhYmxlICVpbiUgYygnbW90aGVyX2FnZScsICdmYXRoZXJfYWdlJykpLCBicmVha3MgPSBzZXEoMCwgNTAsIDIpLCBjbG9zZWQgPSAncmlnaHQnLCBmaWxsID0gZmlsbF9jb2wxLCBjb2xvdXIgPSAnYmxhY2snICkgKwogIGdlb21faGlzdG9ncmFtKGRhdGE9c3Vic2V0KGRmbCwgdmFyaWFibGUgJWluJSBjKCdwcmV2X21hbGVfY29wdWxhdGlvbnMnKSksIGJyZWFrcyA9IHNlcSgwLDQwLDIpLCBjbG9zZWQgPSAncmlnaHQnLCBmaWxsID0gZmlsbF9jb2wyLCBjb2xvdXIgPSAnYmxhY2snICApICsKICBnZW9tX2hpc3RvZ3JhbShkYXRhPXN1YnNldChkZmwsIHZhcmlhYmxlICVpbiUgYygncHJldl9mZW1hbGVfY29wdWxhdGlvbnMnKSksIGJyZWFrcyA9IHNlcSgwLDIwLDEpLCBjbG9zZWQgPSAncmlnaHQnLCBmaWxsID0gZmlsbF9jb2wyLCBjb2xvdXIgPSAnYmxhY2snICApICsKICBnZW9tX2hpc3RvZ3JhbShkYXRhPXN1YnNldChkZmwsIHZhcmlhYmxlICVpbiUgYygna2luJykpLCBicmVha3MgPSBzZXEoMCwgMC4zLCAwLjAxKSwgY2xvc2VkID0gJ3JpZ2h0JywgZmlsbCA9IGZpbGxfY29sMywgY29sb3VyID0gJ2JsYWNrJyAgKSArIAogIGZhY2V0X3dyYXAofnZhcmlhYmxlLCBzY2FsZXM9J2ZyZWUnLCBsYWJlbGxlcj1sYWJlbGxlcih2YXJpYWJsZT1mYWNldGxhYnMpLCBuY29sPTIpICsKICAgIHNjYWxlX3hfY29udGludW91cyhleHBhbmQgPSBjKDAuMDEsMCkpICsKICBsYWJzKHkgPSAnRnJlcXVlbmN5JywgeCA9ICcnKSArIAogIGd1aWRlcyhmaWxsID0gJ25vbmUnKQoKCiBnZ3NhdmUoIi4vZmlndXJlL2ZpZ3VyZV9zMl9wcmVkaWN0b3JfZGlzdHJpYnV0aW9ucy5wZGYiLCBoZWlnaHQgPSA3LCB3aWR0aCA9IDgpCgpgYGAKCmBgYHtyIG1vZGVsX3NldHVwfQojIFJhbmRvbSB2YXJpYWJsZXM6Cmdyb3VwaW5nX3ZhcmlhYmxlcyA8LSBjKCJmYXRoZXIiLCAibW90aGVyIiwgInllYXIiKQoKIyBQcmVkaWN0b3JzOgpwcmVkaWN0b3JzIDwtIGMoCiAgInNjbW90aGVyX2FnZSIsICJoYW5kcmVhcl9tb3RoZXIiLCAic2NmYXRoZXJfYWdlIiwKICAiaGFuZHJlYXJfZmF0aGVyIiwgImNvcHVsYXRpb25zX21hdGVzIiwgInNjcHJldl9tYWxlX2NvcHVsYXRpb25zIiwKICAic2NwcmV2X2ZlbWFsZV9jb3B1bGF0aW9ucyIsICJzY2tpbiIKKQpgYGAKCiMjIFJlZmVyZW5jZSBtb2RlbAoKRml0IGZ1bGwgcmVmZXJlbmNlIG1vZGVsCgpgYGB7ciBtb2RlbF9zdHJ1Y3R1cmV9CiMgRm9ybXVsYSBmb3IgbW9kZWwKZm9ybXVsYSA8LSBwYXN0ZSgKICAiY2x1dGNoX2ZlcnQgfiIsIHBhc3RlMChwcmVkaWN0b3JzLCBjb2xsYXBzZSA9ICIgKyAiKSwgIiArICIsCiAgcGFzdGUocGFzdGUwKCIoMSB8ICIsIGdyb3VwaW5nX3ZhcmlhYmxlcywgIikiKSwgY29sbGFwc2UgPSAiICsgIiksCiAgIisgaGFuZHJlYXJfbW90aGVyOmhhbmRyZWFyX2ZhdGhlciIKKSAlPiUKICBhcy5mb3JtdWxhKCkKYGBgCgpgYGB7ciBvcmlnX2ZpdCwgd2FybmluZ3MgPSBUUlVFfQojIEZpdCBvcmlnaW5hbCBtb2RlbCB3aXRoIHticm1zfQpmaXRfb3JpZ2luYWwgPC0gYnJtKGZvcm11bGEsCiAgZGF0YSA9IGRhdGEsIGZhbWlseSA9IGJlcm5vdWxsaSgpLAogIHByaW9yID0gc2V0X3ByaW9yKGhvcnNlc2hvZSgpKSwKICBpdGVyID0gbml0ZXIsIAogIGNoYWlucyA9IG5jaGFpbnMsCiAgc2VlZCA9IDExMTEsCiAgY29udHJvbCA9IGxpc3QoYWRhcHRfZGVsdGEgPSAwLjk5KQopCmZpdF9vcmlnaW5hbAoKYGBgCgojIyBSZXN0cmljdGVkIHZhcmlhYmxlIHNlbGVjdGlvbgoKYGBge3IgcmVmX21vZGVsfQojIFNldCB1cCB2YXJpYWJsZSBzZWxlY3Rpb24KIyMgcnVuIHZhcmlhYmxlIHNlbGVjdGlvbiBvbiB0aGUgdW5jb25zdHJhaW5lZCBsYXRlbnQgc3BhY2UKcmVmX29yaWdpbmFsIDwtIGJybXM6OjpnZXRfcmVmbW9kZWwuYnJtc2ZpdChmaXRfb3JpZ2luYWwsIHNlZWQgPSAxMTExKQpmX2xhdGVudCA8LSBjb2xNZWFucyhwb3N0ZXJpb3JfbGlucHJlZChmaXRfb3JpZ2luYWwsIHRyYW5zZm9ybSA9IEZBTFNFKSkKZGF0YVtbIi55Il1dIDwtIGZfbGF0ZW50CmRpc19sYXRlbnQgPC0gcmVwKDEsIDIqbml0ZXIpCgojIEdldCByZWZlcmVuY2UgbW9kZWwKcmVmIDwtIGluaXRfcmVmbW9kZWwoZml0X29yaWdpbmFsLAogZGF0YSA9IGRhdGEsIGZvcm11bGEgPSBmb3JtdWxhLCBmYW1pbHkgPSBnYXVzc2lhbigpLAogcmVmX3ByZWRmdW4gPSByZWZfcHJlZGZ1biwgZGl2X21pbmltaXplciA9IHByb2pwcmVkOjo6bGluZWFyX211bHRpbGV2ZWxfbWxlLAogcHJval9wcmVkZnVuID0gcHJvanByZWQ6OjpsaW5lYXJfbXVsdGlsZXZlbF9wcm9qX3ByZWRmdW4sIGRpcyA9IGRpc19sYXRlbnQsCiBleHRyYWN0X21vZGVsX2RhdGEgPSBleHRyYWN0X21vZGVsX2RhdGEsIGN2ZnVuID0gcmVmX29yaWdpbmFsJGN2ZnVuLAogc2VlZCA9IDExMTEKKQpgYGAKCkNvbnN0cmFpbiB0aGUgdmFyaWFibGUgc2VsZWN0aW9uIHRvIHNlbGVjdCB0aGUgZml4ZWQgdGVybXMgZmlyc3QsIHRoZW4gdGhlIHJhbmRvbSB0ZXJtcy4KCmBgYHtyIHJlc3RyaWN0X3NlYXJjaF90ZXJtc30KIyBPcmRlciB2YXJpYWJsZXMgZm9yIHNlbGVjdGlvbjogZmlyc3QgZml4ZWQgdGVybXMsIHRoZW4gcmFuZG9tIHRlcm1zCnNlYXJjaF90ZXJtcyA8LSBjKAogICIxIiwKICAiaGFuZHJlYXJfbW90aGVyICsgaGFuZHJlYXJfZmF0aGVyICsgaGFuZHJlYXJfbW90aGVyOmhhbmRyZWFyX2ZhdGhlciIsCiAgInNjbW90aGVyX2FnZSIsCiAgImhhbmRyZWFyX21vdGhlciIsCiAgInNjZmF0aGVyX2FnZSIsCiAgImhhbmRyZWFyX2ZhdGhlciIsCiAgImNvcHVsYXRpb25zX21hdGVzIiwKICAic2NwcmV2X21hbGVfY29wdWxhdGlvbnMiLAogICJzY3ByZXZfZmVtYWxlX2NvcHVsYXRpb25zIiwKICAic2NraW4iLAogIHBhc3RlKAogICAgInNjbW90aGVyX2FnZSArIGhhbmRyZWFyX21vdGhlciArIHNjZmF0aGVyX2FnZSArIGhhbmRyZWFyX2ZhdGhlciArIiwKICAgICJjb3B1bGF0aW9uc19tYXRlcyArIHNjcHJldl9tYWxlX2NvcHVsYXRpb25zICsgc2NwcmV2X2ZlbWFsZV9jb3B1bGF0aW9ucyArIHNja2luICsiLAogICAgImhhbmRyZWFyX21vdGhlcjpoYW5kcmVhcl9mYXRoZXIgKyAoMSB8IGZhdGhlcikiCiAgKSwKICBwYXN0ZSgKICAgICJzY21vdGhlcl9hZ2UgKyBoYW5kcmVhcl9tb3RoZXIgKyBzY2ZhdGhlcl9hZ2UgKyBoYW5kcmVhcl9mYXRoZXIgKyIsCiAgICAiY29wdWxhdGlvbnNfbWF0ZXMgKyBzY3ByZXZfbWFsZV9jb3B1bGF0aW9ucyArIHNjcHJldl9mZW1hbGVfY29wdWxhdGlvbnMgKyBzY2tpbiArIiwKICAgICJoYW5kcmVhcl9tb3RoZXI6aGFuZHJlYXJfZmF0aGVyICsgKDEgfCBtb3RoZXIpIgogICksCiAgcGFzdGUoCiAgICAic2Ntb3RoZXJfYWdlICsgaGFuZHJlYXJfbW90aGVyICsgc2NmYXRoZXJfYWdlICsgaGFuZHJlYXJfZmF0aGVyICsiLAogICAgImNvcHVsYXRpb25zX21hdGVzICsgc2NwcmV2X21hbGVfY29wdWxhdGlvbnMgKyBzY3ByZXZfZmVtYWxlX2NvcHVsYXRpb25zICsgc2NraW4gKyIsCiAgICAiaGFuZHJlYXJfbW90aGVyOmhhbmRyZWFyX2ZhdGhlciArICgxIHwgeWVhcikiCiAgKQopCmBgYAoKYGBge3IgdmFyc2VsX3Jlc3RyaWN0fQp2c19yZXN0cmljdGVkIDwtIHZhcnNlbChyZWYsIHNlYXJjaF90ZXJtcyA9IHNlYXJjaF90ZXJtcywgc2VlZD0xMTExKQpzdW1tYXJ5KHZzX3Jlc3RyaWN0ZWQsIHN0YXRzID0gYygnZWxwZCcpLCB0eXBlID0gYygnbWVhbicsICdzZScsICdsb3dlcicsICd1cHBlcicsICdkaWZmJywgJ2RpZmZfc2UnKSwgZGVsdGFzID0gVCkKdnNfYmlub20gPC0gdnNfcmVzdHJpY3RlZAoKIyBQcm9qZWN0IG9udG8gbGF0ZW50IHNwYWNlIGFuZCB0aGVuIGJhY2sgdG8gYmlub21pYWw6IGdldCBiZXR0ZXIgcmVzdWx0cyB0aGFuIGlmIGp1c3QgcHJvamVjdGluZyBvcmlnaW5hbCBmdWxsIG1vZGVsLgpsbF9mdW4gPC0gcmVmX29yaWdpbmFsJGZhbWlseSRsbF9mdW4KbGlua2ludiA8LSByZWZfb3JpZ2luYWwkZmFtaWx5JGxpbmtpbnYKZm9yIChrIGluIHNlcV9hbG9uZyh2c19yZXN0cmljdGVkJHN1bW1hcmllcyRzdWIpKSB7CiAgdnNfYmlub20kc3VtbWFyaWVzJHN1Yltba11dJG11IDwtIGxpbmtpbnYodnNfcmVzdHJpY3RlZCRzdW1tYXJpZXMkc3ViW1trXV0kbXUpCiAgdnNfYmlub20kc3VtbWFyaWVzJHN1Yltba11dJGRyYXdzIDwtCiAgICBsaW5raW52KHZzX3Jlc3RyaWN0ZWQkc3VtbWFyaWVzJHN1Yltba11dJGRyYXdzKQogIGxwcGQgPC0gbGxfZnVuKAogICAgbXU9dnNfYmlub20kc3VtbWFyaWVzJHN1Yltba11dJGRyYXdzLAogICAgZGlzPU5VTEwsIHk9YXMubnVtZXJpYyhhcy5tYXRyaXgoZGF0YVssICJjbHV0Y2hfZmVydCJdKSksCiAgICB3ZWlnaHRzID0gcmVmJHdvYnMKICApCiAgdnNfYmlub20kc3VtbWFyaWVzJHN1Yltba11dJGxwcGQgPC0gYXBwbHkoCiAgICBscHBkLCAxLCBwcm9qcHJlZDo6OmxvZ193ZWlnaHRlZF9tZWFuX2V4cCwgcmVwKDEgLyBOQ09MKGxwcGQpLCBOQ09MKGxwcGQpKQogICkKfQoKIyBUcmFuc2Zvcm0gYmFjayB0byBiaW5vbWlhbCBzcGFjZToKdnNfYmlub20kc3VtbWFyaWVzJHJlZiRtdSA8LSBsaW5raW52KHZzX3Jlc3RyaWN0ZWQkc3VtbWFyaWVzJHJlZiRtdSkKdnNfYmlub20kc3VtbWFyaWVzJHJlZiRkcmF3cyA8LSBsaW5raW52KHZzX3Jlc3RyaWN0ZWQkc3VtbWFyaWVzJHJlZiRkcmF3cykKbHBwZCA8LSBsbF9mdW4oCiAgdnNfYmlub20kc3VtbWFyaWVzJHJlZiRkcmF3cywKICBOVUxMLCBhcy5udW1lcmljKGFzLm1hdHJpeChkYXRhWywgImNsdXRjaF9mZXJ0Il0pKSwKICB3ZWlnaHRzID0gcmVmJHdvYnMKKQp2c19iaW5vbSRzdW1tYXJpZXMkcmVmJGxwcGQgPC0gYXBwbHkoCiAgbHBwZCwgMSwgcHJvanByZWQ6Ojpsb2dfd2VpZ2h0ZWRfbWVhbl9leHAsIHJlZiR3c2FtcGxlCikKYGBgCgpgYGB7ciB2YXJzZWxfcGxvdH0KIyMgUGxvdCB2YXJpYWJsZSBzZWxlY3Rpb24gcmVzdWx0cwplbHBkX3JlcGxhY2UgPC0gZnVuY3Rpb24oc3RyaW5nKXsKICBzdHJpbmcgPC0gcGFzdGUodG91cHBlcihzdHJpbmcpLCAiIGRpZmZlcmVuY2UgdnMgcmVmZXJlbmNlIG1vZGVsIikKfQpwbG90KHZzX2Jpbm9tLCBzdGF0cyA9IGMoJ2VscGQnKSwgZGVsdGFzPVQsIGFscGhhPTAuMzIpICsKICBzY2FsZV94X2NvbnRpbnVvdXMoYnJlYWtzID0gYygwLCBzZXFfYWxvbmcodnNfcmVzdHJpY3RlZCRzb2x1dGlvbl90ZXJtcykpLAogICAgICAgICAgICAgICAgICAgICBsYWJlbHMgPSBjKCJJbnRlcmNlcHQiLCBmbl9sYWJlbHModnNfcmVzdHJpY3RlZCRzb2x1dGlvbl90ZXJtcykpKSArCiAgc2NhbGVfeV9jb250aW51b3VzKGV4cGFuZCA9IGMoMCwwKSkgKwogIGV4cGFuZF9saW1pdHMoeT1jKC02MCwxKSkgKwogIGZhY2V0X3dyYXAoc3RhdGlzdGljIH4gLiwgc3RyaXAucG9zaXRpb24gPSAibGVmdCIsIGxhYmVsbGVyID0gbGFiZWxsZXIoc3RhdGlzdGljPWVscGRfcmVwbGFjZSkpICsKICB0aGVtZShheGlzLnRleHQueCA9IGVsZW1lbnRfdGV4dChhbmdsZSA9IDMwLCBoanVzdCA9IDEsIHZqdXN0ID0gMSksCiAgICAgICAgYXhpcy50aWNrcyA9IGVsZW1lbnRfbGluZSgpLAogICAgICAgIHBhbmVsLmdyaWQubWFqb3IueCA9IGVsZW1lbnRfYmxhbmsoKSwKICAgICAgICBwYW5lbC5ncmlkLm1pbm9yLnggPSBlbGVtZW50X2JsYW5rKCksCiAgICAgICAgcGFuZWwuZ3JpZC5tYWpvci55ID0gZWxlbWVudF9ibGFuaygpLAogICAgICAgIHBhbmVsLmdyaWQubWlub3IueSA9IGVsZW1lbnRfYmxhbmsoKSwKICAgICAgICBzdHJpcC50ZXh0ID0gZWxlbWVudF9ibGFuaygpLCAjIG5vIGZhY2V0IHRleHQKICAgICAgICBzdHJpcC5wbGFjZW1lbnQgPSAib3V0c2lkZSIsCiAgICAgICAgc3RyaXAuYmFja2dyb3VuZCA9IGVsZW1lbnRfYmxhbmsoKSwKICAgICAgcGFuZWwuYm9yZGVyID0gZWxlbWVudF9yZWN0KGZpbGwgPSBOQSwgY29sb3VyID0gJ2JsYWNrJykpICsKICBsYWJzKHg9J1Rlcm1zIGluIHRoZSBzdWJtb2RlbCcsIHkgPSAnRUxQRCBkaWZmZXJlbmNlIGZyb20gdGhlIHJlZmVyZW5jZSBtb2RlbCcpIApnZ3NhdmUoZmlsZS5wYXRoKG91dGRpciwiZmlndXJlXzFfdmFyc2VsLnBkZiIpLCBoZWlnaHQgPSA2LCB3aWR0aCA9IDgpCmBgYAoKYGBge3IgdmFyc2VsX3N1bW1hcnksIGNhcHRpb24gPSAiQ29udHJpYnV0aW9uIHRvIGV4cGVjdGVkIGxvZyBwcmVkaWN0aXZlIGRlbnNpdHkgKGVscGQpIGZvciBlYWNoIHRlcm0gaW4gdGhlIG1vZGVsLiBDb2x1bW4gJ2VscGQnIHNob3dzIHRoZSBkaWZmZXJlbmNlIGluIGVscGQgZnJvbSB0aGUgcmVmZXJlbmNlIG1vZGVsOyAnZWxwZF9kaWZmJyBhbmQgJ2VscGRfZGlmZl9wYycgZ2l2ZSB0aGUgZGlmZmVyZW5jZSBhbmQgcGVyY2VudGFnZSBkaWZmZXJlbmNlIGluIHRoZSBlbHBkIGNvbXBhcmVkIHRvIHRoZSBwcmV2aW91cyB0ZXJtLiAifQojIE1ha2VrIHRhYmxlIG9mIHZhcmlhYmxlbCBzZWxlY3Rpb24gcmVzdWx0cwpkIDwtIHN1bW1hcnkodnNfYmlub20sIHR5cGU9YygnbWVhbicsICdzZScsICdkaWZmJywgJ2xvd2VyJywgJ3VwcGVyJyksIHN0YXRzPWMoJ2VscGQnKSwgZGVsdGFzPVQpCgojIEFkZCBjb250cmlidXRpb24gb2YgZGlmZmVyZW5jZSB0byBFTFBEIGZvciBlYWNoIHRlcm0sIGZyb20gdGhlIHByZXZpb3VzIHRlcm06CnRvdF9lbHBkIDwtIGQkc2VsZWN0aW9uJGVscGRbbnJvdyhkJHNlbGVjdGlvbildIC0gZCRzZWxlY3Rpb24kZWxwZFsxXQpkc2VsIDwtIGQkc2VsZWN0aW9uICU+JSBtdXRhdGUoZWxwZF9kaWZmID0gZWxwZCAtIGxhZyhlbHBkKSwKICAgICAgICAgICAgICAgICAgICAgICBlbHBkX2RpZmZfcGMgPSAxMDAgKiBlbHBkX2RpZmYgLyB0b3RfZWxwZCkKZm9ybWF0KGRzZWwsIGRpZ2l0cz0yKQoKIyBSYW5kb20gdGVybSBjb250cmlidXRpb25zIHRvIEVMUEQ6CmNhdChzcHJpbnRmKCJDb250cmlidXRpb24gb2YgcmFuZG9tIG1vZGVsIHRlcm1zIHRvIEVMUEQgZGlmZmVyZW5jZSA9ICUuMWYlJSIsIGRzZWwgJT4lIGZpbHRlcihncmVwbCgifCIsIHNvbHV0aW9uX3Rlcm1zLCBmaXhlZD1UKSkgJT4lIHN1bW1hcmlzZSh0b3RfZWxwZF9kaWZmX3BjID0gc3VtKGVscGRfZGlmZl9wYykpICU+JSBhcy5udW1lcmljKCkpKQpgYGAKCgojIyMgUHJvamVjdGlvbiAtIGZ1bGwgbW9kZWwKClByb2plY3RlZCBtYXJnaW5hbHMgb2YgdGhlIGZ1bGwgbW9kZWwKCmBgYHtyIHByb2plY3RfZnVsbH0KIyBTZXQgdGhlIHNvbHV0aW9uIHRlcm1zIC0gYWxsIHRlcm1zCmlmIChzdWdnZXN0X3NpemUodnNfYmlub20pID4gbGVuZ3RoKHZzX2Jpbm9tJHNvbHV0aW9uX3Rlcm1zKSl7CiAgc29sdXRpb25fdGVybXMgPC0gdnNfYmlub20kc29sdXRpb25fdGVybXNbc2VxX2xlbihzdWdnZXN0X3NpemUodnNfYmlub20pLTEpXQp9IGVsc2UgewogIHNvbHV0aW9uX3Rlcm1zIDwtIHZzX2Jpbm9tJHNvbHV0aW9uX3Rlcm1zW3NlcV9sZW4oc3VnZ2VzdF9zaXplKHZzX2Jpbm9tKSldCn0KcHJvamVjdGlvbiA8LSBwcm9qcHJlZDo6cHJvamVjdCh2c19yZXN0cmljdGVkLCBzb2x1dGlvbl90ZXJtcyA9IHNvbHV0aW9uX3Rlcm1zLCBzZWVkPTExMTEpCgojUHJvamVjdCBvbnRvIGxhdGVudCBzcGFjZSBhbmQgdGhlbiBiYWNrIHRvIGJpbm9taWFsOiBnZXQgYmV0dGVyIHJlc3VsdHMgdGhhbiBpZiBqdXN0IHByb2plY3Rpbmcgb3JpZ2luYWwgZnVsbCBtb2RlbC4KbGxfZnVuIDwtIHJlZl9vcmlnaW5hbCRmYW1pbHkkbGxfZnVuCmxpbmtpbnYgPC0gcmVmX29yaWdpbmFsJGZhbWlseSRsaW5raW52CmZvciAoayBpbiBzZXFfYWxvbmcocHJvamVjdGlvbiRzdW1tYXJpZXMkc3ViKSkgewogIHByb2plY3Rpb24kc3VtbWFyaWVzJHN1Yltba11dJG11IDwtIGxpbmtpbnYocHJvamVjdGlvbiRzdW1tYXJpZXMkc3ViW1trXV0kbXUpCiAgcHJvamVjdGlvbiRzdW1tYXJpZXMkc3ViW1trXV0kZHJhd3MgPC0KICAgIGxpbmtpbnYocHJvamVjdGlvbiRzdW1tYXJpZXMkc3ViW1trXV0kZHJhd3MpCiAgbHBwZCA8LSBsbF9mdW4oCiAgICBwcm9qZWN0aW9uJHN1bW1hcmllcyRzdWJbW2tdXSRkcmF3cywKICAgIE5VTEwsIGFzLm51bWVyaWMoYXMubWF0cml4KGRhdGFbLCAiY2x1dGNoX2ZlcnQiXSkpLAogICAgd2VpZ2h0cyA9IHJlZiR3b2JzCiAgKQogIHByb2plY3Rpb24kc3VtbWFyaWVzJHN1Yltba11dJGxwcGQgPC0gYXBwbHkoCiAgICBscHBkLCAxLCBwcm9qcHJlZDo6OmxvZ193ZWlnaHRlZF9tZWFuX2V4cCwgcmVwKDEgLyBOQ09MKGxwcGQpLCBOQ09MKGxwcGQpKQogICkKfQoKIyBQcm9qZWN0IGJhY2sgdG8gYmlub21pYWwgc3BhY2UKcHJvamVjdGlvbiRzdW1tYXJpZXMkcmVmJG11IDwtIGxpbmtpbnYocHJvamVjdGlvbiRzdW1tYXJpZXMkcmVmJG11KQpwcm9qZWN0aW9uJHN1bW1hcmllcyRyZWYkZHJhd3MgPC0gdnNfYmlub20kc3VtbWFyaWVzJHJlZiRkcmF3cwpscHBkIDwtIGxsX2Z1bigKICBwcm9qZWN0aW9uJHN1bW1hcmllcyRyZWYkZHJhd3MsCiAgTlVMTCwgYXMubnVtZXJpYyhhcy5tYXRyaXgoZGF0YVssICJjbHV0Y2hfZmVydCJdKSksCiAgd2VpZ2h0cyA9IHJlZiR3b2JzCikKcHJvamVjdGlvbiRzdW1tYXJpZXMkcmVmJGxwcGQgPC0gYXBwbHkoCiAgbHBwZCwgMSwgcHJvanByZWQ6Ojpsb2dfd2VpZ2h0ZWRfbWVhbl9leHAsIHJlZiR3c2FtcGxlCikKYGBgCgpSXjIgZm9yIG9yaWdpbmFsIG1vZGVsOgoKYGBge3IgcjJfb3JpZ2luYWx9CiMgQ29tcGFyZSB3aXRoIGJybXM6OmJheWVzX1IyIGZ1bmN0aW9uCmJheWVzX1IyKGZpdF9vcmlnaW5hbCkKYGBgCgpSXjIgZm9yIGZ1bGwgcHJvamVjdGlvbjoKCmBgYHtyIHIyX3Byb2plY3Rpb25fZnVsbH0KIyBNb2RpZmllZCBmdW5jdGlvbiB0byBjYWxjdWxhdGUgUjIgZm9yIHByb2plY3RlZCBtb2RlbAojIE1vZGlmaWVkIGZyb20gaHR0cHM6Ly9hdmVodGFyaS5naXRodWIuaW8vYmF5ZXNfUjIvYmF5ZXNfUjIuaHRtbCMxX0ludHJvZHVjdGlvbgpiYXllc19SMl9yZXMgPC0gZnVuY3Rpb24ocHJvaikgewogIHkgPC0gZ2V0X3kocHJvaiRyZWZtb2RlbCRmaXQpCiAgIyBVc2UgcHJval9saW5wcmVkIGluc3RlYWQgb2YgcnN0YW5hcm06OnBvc3Rlcmlvcl9lcHJlZAogICMgICB1c2UgJHByZWQgdG8gZ2V0IHZhbHVlcyBpbiByZXNwb25zZSBzcGFjZQogIHlwcmVkX2xhdGVudCA8LSBwcm9qX2xpbnByZWQocHJvaiwgdHJhbnNmb3JtID0gVCkkcHJlZAogICMgQXBwbHkgaW52ZXJzZSBsaW5rIGZ1bmN0aW9uIHRvIG91dHB1dCBvZiBwcm9qX2xpbnByZWQgdG8gY29udmVydCBmcm9tIGxhdGVudCBmdW5jdGlvbgogIHlwcmVkIDwtIHJlZl9vcmlnaW5hbCRmYW1pbHkkbGlua2ludih5cHJlZF9sYXRlbnQpCgogIGlmIChwcm9qJHJlZm1vZGVsJGZpdCRmYW1pbHkkZmFtaWx5ID09ICJiaW5vbWlhbCIgJiYgTkNPTCh5KSA9PSAyKSB7CiAgICB0cmlhbHMgPC0gcm93U3Vtcyh5KQogICAgeSA8LSB5WywgMV0KICAgIHlwcmVkIDwtIHlwcmVkICUqJSBkaWFnKHRyaWFscykKICB9CiAgZSA8LSAtMSAqIHN3ZWVwKHlwcmVkLCAyLCB5KQogIHZhcl95cHJlZCA8LSBhcHBseSh5cHJlZCwgMSwgdmFyKQogIHZhcl9lIDwtIGFwcGx5KGUsIDEsIHZhcikKICByMiA8LSB2YXJfeXByZWQgLyAodmFyX3lwcmVkICsgdmFyX2UpCiAgICByMiAlPiUgYXMuZGF0YS5mcmFtZSguKSAlPiUKICAgIHN1bW1hcmlzZShNZWFuID0gbWVhbihyMiksIFNEID0gc2QocjIpLCBRMi41ID0gcXVhbnRpbGUocjIsIDAuMDI1KSwgUTk3LjUgPSBxdWFudGlsZShyMiwgMC45NzUpKQoKfQooUjJfcmVzIDwtIGJheWVzX1IyX3Jlcyhwcm9qZWN0aW9uKSkKCmBgYAoKClJPUEUgcGxvdCBhbmQgcG9zdGVyaW9yIHN0YXRpc3RpY3MuIFVzZSBgcm9wZV9yYW5nZWAgdG8gc3BlY2lmeSByYW5nZSBvZiBST1BFLCB3aGljaCBpcyBgciByb3BlX3JhbmdlKGZpdF9vcmlnaW5hbClgIGZvciBsb2dpc3RpYyByZWdyZXNzaW9uLgoKYGBge3IgcHJvamVjdF9mdWxsX3JvcGUsIGV2YWw9VCwgZmlnLmhlaWdodD04fQojIFBsb3QgUk9QRQptcCA8LSBhcy5kYXRhLmZyYW1lKGFzLm1hdHJpeChwcm9qZWN0aW9uKSkgJT4lIAogIHNlbGVjdCghc3RhcnRzX3dpdGgoInJfIiksIC1zaWdtYSkgIyBkb24ndCBwbG90IHJhbmRvbSBmYWN0b3JzIG9yIHNpZ21hCgojIEFkZCBtZWRpYW4gYW5kIEhESQpnbXAgPC0gcGl2b3RfbG9uZ2VyKG1wICU+JSBzZWxlY3QoLWJfSW50ZXJjZXB0KSwgY29scz1ldmVyeXRoaW5nKCksIG5hbWVzX3RvPSd2YXJpYWJsZScpCgojIFJldGFpbiBvcmRlciBvZiB2YXJpYWJsZXMgZm9yIHBsb3Q6CmdtcCR2YXJpYWJsZSA8LSBmY3RfcmVsZXZlbChnbXAkdmFyaWFibGUsIG5hbWVzKG1wKSkKZ21wJHkgPC0gZ21wJHZhcmlhYmxlCihwIDwtIHBsb3Qocm9wZShtcCxjaT1yb3BlY2ksIHJhbmdlPXJvcGVfcmFuZ2UoZml0X29yaWdpbmFsKSkpICsgCiAgICBzdGF0X3BvaW50aW50ZXJ2YWwoZGF0YT1nbXAsIGFlcyh5PWZhY3Rvcih2YXJpYWJsZSwgbGV2ZWxzPW5hbWVzKG1wKSksIHg9dmFsdWUsIGhlaWdodD1OVUxMLGZpbGw9TlVMTCksIHBvaW50X2ludGVydmFsPW1lZGlhbl9oZGksIC53aWR0aD1jKDAuNSwgMC45NSksIG5vcm1hbGl6ZT0neHknLHBvaW50X3NpemU9MywgcG9pbnRfY29sb3VyPSdibGFjaycsIGludGVydmFsX2NvbG91cj0nYmx1ZScsIHNoYXBlPTIxLCBwb2ludF9maWxsPSd5ZWxsb3cnLCBhbHBoYT0wLjUpICsKICAgIHNjYWxlX3lfZGlzY3JldGUobGFiZWxzPWZuX2xhYmVscywgZXhwYW5kID0gZXhwYW5zaW9uKGFkZCA9IGMoLTAsMS4yKSkpICArCiAgICBzY2FsZV94X2NvbnRpbnVvdXMobGFiZWxzID0gZnVuY3Rpb24oeCl4LzIpICsgIyB0byBjb252ZXJ0IHBvc3RlcmlvciBzY2FsZSBmcm9tIDAuNSBTRCB0byBTRAogICAgbGFicyh0aXRsZT0nJywgeSA9ICdWYXJpYWJsZScsIHggPSAiUG9zc2libGUgdmFsdWVzIChTRCkiKSAgKwogICAgZ3VpZGVzKGZpbGwgPSAnbm9uZScpIAopCgpnZ3NhdmUoZmlsZS5wYXRoKG91dGRpciwgImZpZ3VyZV8yX3Bvc3Rlcmlvcl9mdWxsLnBkZiIpLCBoZWlnaHQgPSA2LCB3aWR0aCA9IDgpCgojIFBvc3RlcmlvciBzdGF0aXN0aWNzCm5hbWVzKG1wKSA8LSBmbl9sYWJlbHMobmFtZXMobXApKQpkZXNjcmliZV9wb3N0ZXJpb3IobXAsIGNpX21ldGhvZD0naGRpJywgY2k9MC45NSwgcm9wZV9jaT1yb3BlY2ksIHJvcGVfcmFuZ2U9cm9wZV9yYW5nZShmaXRfb3JpZ2luYWwpLCBjZW50cmFsaXR5ID0gIm1lZGlhbiIsdGVzdCA9IGMoInBfZGlyZWN0aW9uIiwgInBfc2lnbmlmaWNhbmNlIiwgInJvcGUiLCAnZXF1aXZhbGVuY2VfdGVzdCcpKQpgYGAKCiMjIyBQcm9qZWN0aW9uIC0gcmVkdWNlZCBtb2RlbAoKUHJvamVjdCBvbmx5IHRoZSBgY29wdWxhdGlvbnNgIGFuZCBgaGFuZC1yZWFyaW5nYCBmaXhlZCBwcmVkaWN0b3JzLCBzaW5jZSB0aGVzZSBjb250cmlidXRlIHRoZSBtb3N0IHRvIHRoZSB2YXJpYW5jZSBhbmQgaGF2ZSBub24temVybyBwb3N0ZXJpb3JzIGluIHRoZSBmdWxsIG1vZGVsLCBwbHVzIHRoZSByYW5kb20gdGVybXMKCmBgYHtyIHByb2plY3RfcmVkdWNlZCwgZXZhbD1ULCBjYWNoZT1GfQojIFNldCBzb2x1dGlvbiB0ZXJtczogYmVzdCB0d28gZml4ZWQgdGVybXMgYW5kIGFsbCByYW5kb20gdGVybXMKc29sdXRpb25fdGVybXMgPC0gYyh2c19iaW5vbSRzb2x1dGlvbl90ZXJtc1sxOjJdLAogICAgICAgICAgICAgICAgICAgICB2c19iaW5vbSRzb2x1dGlvbl90ZXJtc1sxMDoxMl0pCiAgcHJvamVjdGlvbiA8LSBwcm9qcHJlZDo6cHJvamVjdCh2c19yZXN0cmljdGVkLCBzb2x1dGlvbl90ZXJtcyA9IHNvbHV0aW9uX3Rlcm1zLCBzZWVkPTExMTEpCgojIFByb2plY3Qgb250byBsYXRlbnQgc3BhY2UgYW5kIHRoZW4gYmFjayB0byBiaW5vbWlhbDogZ2V0IGJldHRlciByZXN1bHRzIHRoYW4gaWYganVzdCBwcm9qZWN0aW5nIG9yaWdpbmFsIGZ1bGwgbW9kZWwuCmxsX2Z1biA8LSByZWZfb3JpZ2luYWwkZmFtaWx5JGxsX2Z1bgpsaW5raW52IDwtIHJlZl9vcmlnaW5hbCRmYW1pbHkkbGlua2ludgpmb3IgKGsgaW4gc2VxX2Fsb25nKHByb2plY3Rpb24kc3VtbWFyaWVzJHN1YikpIHsKICBwcm9qZWN0aW9uJHN1bW1hcmllcyRzdWJbW2tdXSRtdSA8LSBsaW5raW52KHByb2plY3Rpb24kc3VtbWFyaWVzJHN1Yltba11dJG11KQogIHByb2plY3Rpb24kc3VtbWFyaWVzJHN1Yltba11dJGRyYXdzIDwtCiAgICBsaW5raW52KHByb2plY3Rpb24kc3VtbWFyaWVzJHN1Yltba11dJGRyYXdzKQogIGxwcGQgPC0gbGxfZnVuKAogICAgcHJvamVjdGlvbiRzdW1tYXJpZXMkc3ViW1trXV0kZHJhd3MsCiAgICBOVUxMLCBhcy5udW1lcmljKGFzLm1hdHJpeChkYXRhWywgImNsdXRjaF9mZXJ0Il0pKSwKICAgIHdlaWdodHMgPSByZWYkd29icwogICkKICBwcm9qZWN0aW9uJHN1bW1hcmllcyRzdWJbW2tdXSRscHBkIDwtIGFwcGx5KAogICAgbHBwZCwgMSwgcHJvanByZWQ6Ojpsb2dfd2VpZ2h0ZWRfbWVhbl9leHAsIHJlcCgxIC8gTkNPTChscHBkKSwgTkNPTChscHBkKSkKICApCn0KCiMgUHJvamVjdCBiYWNrIHRvIGJpbm9taWFsCnByb2plY3Rpb24kc3VtbWFyaWVzJHJlZiRtdSA8LSBsaW5raW52KHByb2plY3Rpb24kc3VtbWFyaWVzJHJlZiRtdSkKcHJvamVjdGlvbiRzdW1tYXJpZXMkcmVmJGRyYXdzIDwtIHZzX2Jpbm9tJHN1bW1hcmllcyRyZWYkZHJhd3MKbHBwZCA8LSBsbF9mdW4oCiAgcHJvamVjdGlvbiRzdW1tYXJpZXMkcmVmJGRyYXdzLAogIE5VTEwsIGFzLm51bWVyaWMoYXMubWF0cml4KGRhdGFbLCAiY2x1dGNoX2ZlcnQiXSkpLAogIHdlaWdodHMgPSByZWYkd29icwopCnByb2plY3Rpb24kc3VtbWFyaWVzJHJlZiRscHBkIDwtIGFwcGx5KAogIGxwcGQsIDEsIHByb2pwcmVkOjo6bG9nX3dlaWdodGVkX21lYW5fZXhwLCByZWYkd3NhbXBsZQopCmBgYAoKUjIgb2YgcHJvamVjdGVkIHJlZHVjZWQgbW9kZWw6CgpgYGB7ciByMl9wcm9qZWN0aW9uX3JlZHVjZWR9CihSMl9yZXMgPC0gYmF5ZXNfUjJfcmVzKHByb2plY3Rpb24pKQpgYGAKCgpST1BFIHBsb3QgYW5kIHBvc3RlcmlvciBzdGF0aXN0aWNzOgoKYGBge3IgcHJvamVjdF9yZWR1Y2VkX3JvcGUsIGV2YWw9VCwgZmlnLmhlaWdodD04fQojIFBsb3QgUk9QRQptcCA8LSBhcy5kYXRhLmZyYW1lKGFzLm1hdHJpeChwcm9qZWN0aW9uKSklPiUgCiAgc2VsZWN0KCFzdGFydHNfd2l0aCgicl8iKSwgLXNpZ21hKSAjIGRvbid0IHBsb3QgcmFuZG9tIGZhY3RvcnMgb3Igc2lnbWEKCiMgQWRkIG1lZGlhbiBhbmQgSERJCmdtcCA8LSBwaXZvdF9sb25nZXIobXAgJT4lIHNlbGVjdCgtYl9JbnRlcmNlcHQpLCBjb2xzPWV2ZXJ5dGhpbmcoKSwgbmFtZXNfdG89J3ZhcmlhYmxlJykKCiMgUmV0YWluIG9yZGVyIG9mIHZhcmlhYmxlczoKZ21wJHZhcmlhYmxlIDwtIGZjdF9yZWxldmVsKGdtcCR2YXJpYWJsZSwgbmFtZXMobXApKQpnbXAkeSA8LSBnbXAkdmFyaWFibGUKcGxvdChyb3BlKG1wLGNpPXJvcGVjaSwgcmFuZ2U9cm9wZV9yYW5nZShmaXRfb3JpZ2luYWwpKSkgKyAKICBzdGF0X3BvaW50aW50ZXJ2YWwoZGF0YT1nbXAsIGFlcyh5PWZhY3Rvcih2YXJpYWJsZSwgbGV2ZWxzPW5hbWVzKG1wKSksIHg9dmFsdWUsIGhlaWdodD1OVUxMLGZpbGw9TlVMTCksIHBvaW50X2ludGVydmFsPW1lZGlhbl9oZGksIC53aWR0aD1jKDAuNSwgMC45NSksIG5vcm1hbGl6ZT0neHknLHBvaW50X3NpemU9MywgcG9pbnRfY29sb3VyPSdibGFjaycsIGludGVydmFsX2NvbG91cj0nYmx1ZScsIHNoYXBlPTIxLHBvaW50X2ZpbGw9J3llbGxvdycsIGFscGhhPTAuNSkgKwogIHNjYWxlX3lfZGlzY3JldGUobGFiZWxzPWZuX2xhYmVscywgZXhwYW5kID0gZXhwYW5zaW9uKGFkZCA9IGMoLTAsMS4yKSkpICsgCiAgICAgIHNjYWxlX3hfY29udGludW91cyhsYWJlbHMgPSBmdW5jdGlvbih4KXgvMikgKyAjIHRvIGNvbnZlcnQgcG9zdGVyaW9yIHNjYWxlIGZyb20gMC41IFNEIHRvIFNECiAgICBsYWJzKHRpdGxlPScnLCB5ID0gJ1ZhcmlhYmxlJywgeCA9ICJQb3NzaWJsZSB2YWx1ZXMgKFNEKSIpICArCiAgZ3VpZGVzKGZpbGw9J25vbmUnKQoKZ2dzYXZlKGZpbGUucGF0aChvdXRkaXIsICJmaWd1cmVfM19wb3N0ZXJpb3JfcmVkdWNlZC5wZGYiKSwgaGVpZ2h0ID0gNiwgd2lkdGggPSA4KQoKIyBQb3N0ZXJpb3Igc3RhdHM6Cm5hbWVzKG1wKSA8LSBmbl9sYWJlbHMobmFtZXMobXApKQpkZXNjcmliZV9wb3N0ZXJpb3IobXAsIGNpX21ldGhvZD0naGRpJywgY2k9MC45NSwgcm9wZV9jaT1yb3BlY2ksIHJvcGVfcmFuZ2U9cm9wZV9yYW5nZShmaXRfb3JpZ2luYWwpLCBjZW50cmFsaXR5ID0gIm1lZGlhbiIsdGVzdCA9IGMoInBfZGlyZWN0aW9uIiwgInBfc2lnbmlmaWNhbmNlIiwgInJvcGUiLCAnZXF1aXZhbGVuY2VfdGVzdCcpKQoKYGBgCgojIyMjIE1hcmdpbmFsIG1lYW5zCgpFdmFsdWF0ZSBpbnRlcmFjdGlvbiBwbG90IG9mIGVzdGltYXRlZCBtYXJnaW5hbCBtZWFucyBmcm9tIHRoZSBwcm9qZWN0ZWQgcG9zdGVyaW9yIGZvciBoYW5kLXJlYXJpbmcgYW5kIG51bWJlciBvZiBjb3B1bGF0aW9ucyBjb21iaW5lZDoKCmBgYHtyIHByb2plY3RfcmVkdWNlZF9tYXJnX2hyX21hdGV9CgojIENyZWF0ZSBwcm9qZWN0aW9uIGRhdGFmcmFtZQptcGIgPC0gYXMuZGF0YS5mcmFtZShhcy5tYXRyaXgocHJvamVjdGlvbikpICU+JSBzZWxlY3Qoc3RhcnRzX3dpdGgoImJfIikpICU+JSByZW5hbWVfd2l0aCguZm49ZnVuY3Rpb24oeCkgZ3N1YigiYl8iLCIiLHgpLCAuY29scz1ldmVyeXRoaW5nKCkpICAlPiUgYXMubWF0cml4KC4pCgojIEdyaWQgZm9yIGNvcHVsYXRpb25zX21hdGVzCmdyZF9ocjIgPC0gcWRyZyh+IGNvcHVsYXRpb25zX21hdGVzICsgaGFuZHJlYXJfZmF0aGVyICwgZGF0YT1maXRfb3JpZ2luYWwkZGF0YSwgbWNtYz1tcGJbLCBjKCdJbnRlcmNlcHQnLCdjb3B1bGF0aW9uc19tYXRlczIrIGNvcHVsYXRpb25zJywgJ2NvcHVsYXRpb25zX21hdGVzRGlmZmVyZW50IG1hbGVzJywgJ2hhbmRyZWFyX2ZhdGhlclRSVUUnKV0sIGxpbms9J2xvZ2l0JykKCgojIFBsb3Qgd2l0aG91dCBkYXRhOgpwcm9qLmludCA8LSBlbW1pcChncmRfaHIyLCBmb3JtdWxhKCJ+Y29wdWxhdGlvbnNfbWF0ZXMgICsgaGFuZHJlYXJfZmF0aGVyIiksIHR5cGU9J3Jlc3BvbnNlJywgcGxvdGl0PUYpICU+JQogIG11dGF0ZShmYXRoZXJfcmVhcj0gY2FzZV93aGVuKGhhbmRyZWFyX2ZhdGhlcj09J1RSVUUnIH4gJ0hhbmQtcmVhcmVkJywKICAgICAgICAgICAgICAgICAgICAgICAgICAgICAgICAgaGFuZHJlYXJfZmF0aGVyPT0nRkFMU0UnIH4gJ1dpbGQtcmVhcmVkJykpCmdocl9tYXRlIDwtIGdncGxvdCgpICsgCiAgZ2VvbV9wb2ludHJhbmdlKGRhdGE9cHJvai5pbnQsIGFlcyh4PWNvcHVsYXRpb25zX21hdGVzLCB5PXl2YXIsIHltaW49TENMLCB5bWF4PVVDTCwgY29sb3VyPWZhdGhlcl9yZWFyLCBzaGFwZSA9IGZhdGhlcl9yZWFyKSwgcG9zaXRpb249cG9zaXRpb25fZG9kZ2Uod2lkdGg9MC4xKSkgICsgCiAgbGFicyh5PSdQcm9iYWJpbGl0eSBvZiBjbHV0Y2ggZmVydGlsaXR5JywgeD0nJywgY29sb3VyPSdGYXRoZXIgcmVhcmluZycsIHNoYXBlID0gJ0ZhdGhlciByZWFyaW5nJykgKyAKICBzY2FsZV94X2Rpc2NyZXRlKGxhYmVscz1mbl9sYWJlbHMpICsKICBzY2FsZV9jb2xvdXJfYnJld2VyKHBhbGV0dGUgPSAnU2V0MicsIGRpcmVjdGlvbiA9IC0xKSAjIGNvbG91ci1ibGluZCBmcmllbmRseQoKZ2dzYXZlKGZpbGUucGF0aChvdXRkaXIsICJmaWd1cmVfNF9wcmVkaWN0LnBkZiIpLCBnaHJfbWF0ZSwgaGVpZ2h0ID0gNiwgd2lkdGggPSA4KQoKCiMgUGxvdCB3aXRoIGRhdGE6Cmdocl9tYXRlICsgCiAgZ2VvbV9qaXR0ZXIoZGF0YT1maXRfb3JpZ2luYWwkZGF0YSAlPiUgCiAgICAgICAgICAgICAgICBtdXRhdGUoZmF0aGVyX3JlYXI9IGNhc2Vfd2hlbigKICAgICAgICAgICAgICAgICAgaGFuZHJlYXJfZmF0aGVyPT0nVFJVRScgfiAnSGFuZC1yZWFyZWQnLAogICAgICAgICAgICAgICAgICBoYW5kcmVhcl9mYXRoZXI9PSdGQUxTRScgfiAnV2lsZC1yZWFyZWQnKSksIAogICAgICAgICAgICAgIGFlcyh4PWNvcHVsYXRpb25zX21hdGVzLCB5PWNsdXRjaF9mZXJ0LCBjb2xvdXI9ZmF0aGVyX3JlYXIsIHNoYXBlID0gZmF0aGVyX3JlYXIpLCBhbHBoYT0wLjUsIHdpZHRoPTAuMSwgaGVpZ2h0PTAuMDUpCmdnc2F2ZShmaWxlLnBhdGgob3V0ZGlyLCAiZmlndXJlXzRfcHJlZGljdF9kYXRhLnBkZiIpLCBoZWlnaHQgPSA2LCB3aWR0aCA9IDgpCgojIFJlc3VsdHMgdGFibGU6CnByb2ouaW50ICU+JSByZW5hbWUoY2x1dGNoLmZlcnRpbGl0eSA9IHl2YXIpICU+JQogIG11dGF0ZShmYXRoZXJfcmVhcj0gY2FzZV93aGVuKGhhbmRyZWFyX2ZhdGhlcj09J1RSVUUnIH4gJ0hhbmQtcmVhcmVkJywKICAgICAgICAgICAgICAgICAgICAgICAgICAgICAgICAgaGFuZHJlYXJfZmF0aGVyPT0nRkFMU0UnIH4gJ1dpbGQtcmVhcmVkJykpICU+JQogIHNlbGVjdChjb3B1bGF0aW9uc19tYXRlcywgZmF0aGVyX3JlYXIsIGNsdXRjaC5mZXJ0aWxpdHksIExDTCwgVUNMKSAlPiUKICBmb3JtYXQoLiwgZGlnaXRzPTMpCmBgYAoKIyBNdWx0aXBsZSBjb3B1bGF0aW9uIGFuZCBrYWthcG8gZGVuc2l0eQoKYGBge3IgZGVuc2l0eV9kYXRhfQojIFJlYWQgbnVtYmVyIG9mIGtha2FwbyBwZXIgaXNsYW5kIHBlciB5ZWFyCm5rIDwtIHJlYWQuY3N2KGtha2Fwb19kZW1vZ19maWxlLCBoZWFkZXIgPSBUKSAKCiMgTnVtYmVyIG9mIGNvcHVsYXRpb25zL21hbGVzIHBlciBpc2xhbmQgcGVyIHllYXIgcGVyIG51bWJlciBvZiBtYXRpbmdzL21hdGVzCnRjIDwtIHRhYmxlKGZ1bGxfZGF0YSRJc2xhbmQsIGZ1bGxfZGF0YSR5ZWFyLCBmdWxsX2RhdGEkY29wdWxhdGlvbnNfbWF0ZXMpCmR0YyA8LSBkYXRhLmZyYW1lKHRjKSAlPiUgcmVuYW1lKElzbGFuZCA9IFZhcjEsIHllYXIgPSBWYXIyLCBjb3B1bGF0aW9ucyA9IFZhcjMpICU+JSBtdXRhdGUoeWVhciA9IGFzLm51bWVyaWMoYXMuY2hhcmFjdGVyKHllYXIpKSkKCiMgVG90YWwgY2x1dGNoZXMgcGVyIGlzbGFuZDoKZHRjX3RvdCA8LSBkdGMgJT4lIGdyb3VwX2J5KHllYXIsIElzbGFuZCkgJT4lIHN1bW1hcmlzZSh0b3RjbHV0Y2g9c3VtKEZyZXEpKQpkdGMgPC0gbGVmdF9qb2luKGR0YywgZHRjX3RvdCwgYnk9YygneWVhcicsICdJc2xhbmQnKSkKCiMgQ29tYmluZSBkYXRhZnJhbWVzOiBudW1iZXIgb2Yga2FrYXBvIHBlciBpc2xhbmQgYW5kIGNvcHVsYXRpb25zIHByb3BvcnRpb25zCm5rMiA8LSBsZWZ0X2pvaW4oZHRjLCBuayAlPiUgZmlsdGVyKGFnZUNsYXNzPT0nQWR1bHQnKSwgYnk9YygneWVhcicsICdJc2xhbmQnKSwgc3VmZml4ID0gYygnLmNvcCcsICcua2FrJykpICU+JQogIG11dGF0ZShwcm9wLmthayA9IEZyZXEuY29wIC8gRnJlcS5rYWssCiAgICAgICAgIHByb3AuY2x1dGNoID0gRnJlcS5jb3AgLyB0b3RjbHV0Y2gpICU+JQogIGZpbHRlcighaXMubmEoU2V4KSAmICFpcy5uYShGcmVxLmthaykgJiBGcmVxLmthayA+IDApCmBgYAoKQ29tYmluZSByZXBlYXRlZCBjb3B1bGF0aW9ucyB3aXRoIHRoZSBzYW1lIG1hbGUgYW5kIGNvcHVsYXRpb25zIHdpdGggZGlmZmVyZW50IG1hbGVzIHRvIGNvbXBhcmUgc2luZ2xlIHZzIG11bHRpcGxlIGNvcHVsYXRpb25zLgoKT25seSBjb25zaWRlciBXaGVudWEgSG91IGZyb20gMTk5MCBvbndhcmRzLgoKYGBge3IgZGVuc2l0eV9jb21iaW5lLCBmaWcud2lkdGg9MTgsIGZpZy5oZWlnaHQ9MTJ9CgpuazIgPC0gbmsyICU+JSBtdXRhdGUoY29wdWxhdGlvbnNfY29tYiA9IGZjdF9jb2xsYXBzZShjb3B1bGF0aW9ucywgCiAgICAgICAgICAgICAgICAgICAgICAgICAgICAgICAgICAgICAgICAgICAgICBzaW5nbGUgPSAiMSBjb3B1bGF0aW9uIiwKICAgICAgICAgICAgICAgICAgICAgICAgICAgICAgICAgICAgICAgICAgICAgIG11bHRpcGxlID0gYygiMisgY29wdWxhdGlvbnMiLCAiRGlmZmVyZW50IG1hbGVzIikpKQojIFRvdGFsczoKbmtnIDwtIG5rMiAlPiUgCiAgZ3JvdXBfYnkoSXNsYW5kLCB5ZWFyLCBTZXgsIGNvcHVsYXRpb25zX2NvbWIpICU+JSAKICBzdW1tYXJpc2UoRnJlcS5jb3A9c3VtKEZyZXEuY29wLCBuYS5ybT1UKSwgRnJlcS5rYWsgPSBtZWFuKEZyZXEua2FrLCBuYS5ybSA9IFQpLCB0b3RjbHV0Y2ggPSBtZWFuKHRvdGNsdXRjaCwgbmEucm0gPSBUKSkgJT4lCiAgbXV0YXRlKHByb3AuY2x1dGNoID0gRnJlcS5jb3AgLyB0b3RjbHV0Y2gpCgojIE9ubHkgV2hlbnVhIEhvdSBmcm9tIDE5OTA6Cm5rZ3N1YiA8LSBua2cgJT4lIGZpbHRlcihJc2xhbmQ9PSdXaGVudWEgSG91JyAmIHllYXI+PTE5OTAgJiBjb3B1bGF0aW9uc19jb21iID09ICdtdWx0aXBsZScpCmBgYAoKIyMgTnVtYmVyIG9mIGNvcHVsYXRpb25zCgpTaG93IHRhYmxlIG51bWJlciBvZiBjb3B1bGF0aW9ucyB2cyBudW1iZXIgb2YgbWF0ZXMKCmBgYHtyIHRhYl9udW1fY29wX21hdGV9CnRhYmxlKGBOdW1iZXIgb2YgbWF0ZXNgID0gZnVsbF9kYXRhJG51bWJlcl9vZl9tYWxlcywgYE51bWJlciBvZiBjb3B1bGF0aW9uc2AgPSBmdWxsX2RhdGEkbnVtYmVyX29mX2NvcHVsYXRpb25zKQpgYGAKCgojIyBOdW1iZXIgb2Yga2FrYXBvCgpNdWx0aXBsZSBjb3B1bGF0aW9uIHByb3BvcnRpb24gdnMgbnVtYmVyIG9mIG1hbGVzIGFuZCBmZW1hbGVzCgpgYGB7ciBkZW5zaXR5X211bHRfbnVtfQojIENhbGN1bGF0ZSBjb3JyZWxhdGlvbjoKKGNvciA8LSBua2dzdWIgJT4lIHVuZ3JvdXAoLikgJT4lIGRwbHlyOjpzZWxlY3QoU2V4LCBGcmVxLmthaywgcHJvcC5jbHV0Y2gpICU+JSBncm91cF9ieSggU2V4KSAlPiUgY29ycmVsYXRpb24oKSkKY29yJHAKIyBCYXNlIHBsb3Q6CmNvbCA8LSBicmV3ZXIucGFsKG5hbWU9J1NldDEnLG49MylbMl0KZ3doMTk5MF9jb21iIDwtIGdncGxvdChua2dzdWIgJT4lIGZpbHRlcighaXMubmEocHJvcC5jbHV0Y2gpKSwgYWVzKHg9IEZyZXEua2FrLCB5ID0gcHJvcC5jbHV0Y2gpKSArIAogIGdlb21fcG9pbnQoc2l6ZT0yLCBjb2xvdXI9Y29sKSArIAogIGdlb21fc21vb3RoKGFlcyhzaGFwZT1OVUxMKSwgZmlsbD1jb2wsIG1ldGhvZD1sbSwgYWxwaGE9MC4yKSArIAogIGxhYnMoeSA9ICJQcm9wb3J0aW9uIG9mIGNsdXRjaGVzIHdpdGggbXVsdGlwbGUgY29wdWxhdGlvbnMiLCB4ID0gJ051bWJlciBvZiBhZHVsdCBrYWthcG8nKSAgKyAKICBmYWNldF93cmFwKH5TZXgsIHNjYWxlcz0nZnJlZV94JykKCmBgYAoKIyMgU2V4IHJhdGlvCgpNdWx0aXBsZSBjb3B1bGF0aW9uIHByb3BvcnRpb24gdnMgc2V4IHJhdGlvOgoKYGBge3IgZGVuc2l0eV9tdWx0X3NleF9yYXRpb19kYXRhfQojIENhbGN1bGF0ZSBzZXggcmF0aW8Kc3JnIDwtIG5rZ3N1YiAlPiUgCiAgcGl2b3Rfd2lkZXIoaWRfY29scyA9IGMoSXNsYW5kLCB5ZWFyLCBjb3B1bGF0aW9uc19jb21iLCBGcmVxLmNvcCwgdG90Y2x1dGNoLCBwcm9wLmNsdXRjaCksIG5hbWVzX2Zyb20gPSAnU2V4JywgdmFsdWVzX2Zyb209IkZyZXEua2FrIikgJT4lCiAgbXV0YXRlKHNleC5yYXRpbyA9IEZlbWFsZS9NYWxlKQoKIyBDYWxjdWxhdGlvbiBjb3JyZWxhdGlvbjoKKGNvcnNleCA8LSBzcmcgJT4lIHVuZ3JvdXAoLikgJT4lIGRwbHlyOjpzZWxlY3Qoc2V4LnJhdGlvLCBwcm9wLmNsdXRjaCkgICU+JSBjb3JyZWxhdGlvbihiYXllc2lhbj1GKSkKY29yc2V4JHAKYGBgCgpgYGB7ciBkZW5zaXR5X211bHRfc2V4X3JhdGlvLCBmaWcua2VlcD0xfQpjb2wgPC0gYnJld2VyLnBhbChuYW1lPSdTZXQxJyxuPTMpWzFdCiMgUGxvdCBvZiBtdWx0aXBsZSBjb3B1bGF0aW9uIGZyZXF1ZW5jeSB2cyBzZXggcmF0aW8KZ3N3aDE5OTBjb21iIDwtIGdncGxvdChzcmcgJT4lIGZpbHRlcihjb3B1bGF0aW9uc19jb21iPT0nbXVsdGlwbGUnKSwgYWVzKHg9IHNleC5yYXRpbywgeT1wcm9wLmNsdXRjaCkpICsgCiAgZ2VvbV9wb2ludChzaXplPTIsIGNvbG91cj1jb2wpICsgZ2VvbV9zbW9vdGgoYWVzKHNoYXBlPU5VTEwpLCBtZXRob2Q9bG0sIGFscGhhPTAuMiwgY29sb3VyPWNvbCwgZmlsbD1jb2wpICsgCiAgbGFicyh5ID0gIlByb3AuIG9mIGNsdXRjaGVzIHdpdGggbXVsdGlwbGUgY29wdWxhdGlvbnMiLCB4ID0gJ0ZlbWFsZTptYWxlIHNleCByYXRpbycpIAoKYGBgCgojIyBOdW1iZXIgYW5kIHNleCByYXRpbyBjb21iaW5lZAoKQ29tYmluZSBudW1iZXIgb2YgbWFsZS9mZW1hbGUgYW5kIHNleCByYXRpbyBwbG90czoKCmBgYHtyIGNvbWJpbmVfbnVtYmVyX3NleF9yYXRpb30KIyBTZXBhcmF0ZSBwYW5lbHMgZm9yIGZlbWFsZXMsIG1hbGVzIGFuZCBzZXggcmF0aW8KIyBNdWx0aXBsZSBjb3B1bGF0aW9uIGZyZXF1ZW5jeSB2cyBudW1iZXIgb2YgZmVtYWxlcwpjb2wgPC0gYnJld2VyLnBhbChuYW1lPSdTZXQyJyxuPTMpWzFdICMgY29sb3VyLWJsaW5kIGZyaWVuZGx5Cmd3aDE5OTBfZmVtYWxlIDwtIGdncGxvdChua2dzdWIgJT4lIGZpbHRlcighaXMubmEocHJvcC5jbHV0Y2gpICYgU2V4ID09ICdGZW1hbGUnKSwgYWVzKHg9IEZyZXEua2FrLCB5ID0gcHJvcC5jbHV0Y2gpKSArIAogIGdlb21fcG9pbnQoc2l6ZT0yLCBjb2xvdXI9Y29sKSArIAogIGdlb21fc21vb3RoKGFlcyhzaGFwZT1OVUxMKSwgY29sb3VyID0gY29sLCBmaWxsPWNvbCwgbWV0aG9kPWxtLCBhbHBoYT0wLjIpICsgCiAgbGFicyh5ID0gIlByb3BvcnRpb24gb2YgY2x1dGNoZXMiLCB4ID0gJ051bWJlciBvZiBmZW1hbGVzJykgKwogIGNvb3JkX2NhcnRlc2lhbih5bGltID0gYygwLDEpKSArIAogIGV4cGFuZF9saW1pdHMoeD00MCkKCiMgTXVsdGlwbGUgY29wdWxhdGlvbiBmcmVxdWVuY3kgdnMgbnVtYmVyIG9mIG1hbGVzCmNvbCA8LSBicmV3ZXIucGFsKG5hbWU9J1NldDInLG49MylbMl0KZ3doMTk5MF9tYWxlIDwtIGdncGxvdChua2dzdWIgJT4lIGZpbHRlcighaXMubmEocHJvcC5jbHV0Y2gpICYgU2V4ID09ICdNYWxlJyksIGFlcyh4PSBGcmVxLmthaywgeSA9IHByb3AuY2x1dGNoKSkgKyAKICBnZW9tX3BvaW50KHNpemU9MiwgY29sb3VyPWNvbCkgKyAKICBnZW9tX3Ntb290aChhZXMoc2hhcGU9TlVMTCksIGZpbGw9Y29sLCBtZXRob2Q9bG0sIGFscGhhPTAuMiwgY29sb3VyPWNvbCwgZmlsbD1jb2wpICsgCiAgbGFicyh5ID0gIiIsIHggPSAnTnVtYmVyIG9mIG1hbGVzJykgICsKICBjb29yZF9jYXJ0ZXNpYW4oeWxpbSA9IGMoMCwxKSkgKyAKICBleHBhbmRfbGltaXRzKHg9YygxNSwzMCkpCgojIE11bHRpcGxlIGNvcHVsYXRpb24gZnJlcXVlbmN5IHZzIHNleCByYXRpbwpjb2wgPC0gYnJld2VyLnBhbChuYW1lPSdTZXQyJyxuPTMpWzNdCmd3aDE5OTBfc2V4cmF0aW8gPC0gZ2dwbG90KHNyZyAlPiUgZmlsdGVyKGNvcHVsYXRpb25zX2NvbWI9PSdtdWx0aXBsZScpLCBhZXMoeD0gc2V4LnJhdGlvLCB5PXByb3AuY2x1dGNoKSkgKyAKICBnZW9tX3BvaW50KHNpemU9MiwgY29sb3VyPWNvbCkgKyAKICBnZW9tX3Ntb290aChhZXMoc2hhcGU9TlVMTCksIG1ldGhvZD1sbSwgYWxwaGE9MC4yLCBjb2xvdXI9Y29sLCBmaWxsPWNvbCkgKyAKICBsYWJzKHkgPSAiIiwgeCA9ICdGZW1hbGU6bWFsZSBzZXggcmF0aW8nKSArCiAgY29vcmRfY2FydGVzaWFuKHlsaW0gPSBjKDAsMSkpICsgCiAgZXhwYW5kX2xpbWl0cyh4PTAuNCkgCgpnZ2FycmFuZ2UoZ3doMTk5MF9mZW1hbGUsCiAgICAgICAgICBnd2gxOTkwX21hbGUsCiAgICAgICAgICBnd2gxOTkwX3NleHJhdGlvLAogICAgICAgICAgY29tbW9uLmxlZ2VuZCA9IFQgLCAKICAgICAgICAgIGxhYmVscyA9IGMoIkEiLCAiQiIsICJDIiksCiAgICAgICAgICBsYWJlbC54ID0gMC4yNSwKICAgICAgICAgIGxhYmVsLnkgPSAwLjk4LAogICAgICAgICAgbmNvbCA9IDMsCiAgICAgICAgICBucm93ID0gMSwKICAgICAgICAgIHdpZHRocz1jKDEsMSwxKSkgCgoKIyBGb3IgcHVibGljYXRpb246Cmdnc2F2ZShmaWxlbmFtZSA9IGZpbGUucGF0aChvdXRkaXIsICdmaWd1cmVfNV9jb3B1bGF0aW9uX3NleF9yYXRpby5wZGYnKSwgcGxvdD1sYXN0X3Bsb3QoKSwgZGV2aWNlID0gJ3BkZicsIHdpZHRoPTEwLCBoZWlnaHQ9NiApCgpgYGAKCiMjIENoYW5nZSBvdmVyIHRpbWUKClNob3cgdGhlIGNoYW5nZSBpbiBXaGVudWEgSG91IHBvcHVsYXRpb24gYW5kIHNleCByYXRpbyBvdmVyIHRpbWUgKG5vdGUgdGhhdCB0aGVzZSBkYXRhIGluY2x1ZGUgc29tZSB5ZWFycyBpbiB3aGljaCB0aGVyZSB3YXMgbm8gYnJlZWRpbmcpOgoKYGBge3IgdGltZV9zZXhfcmF0aW99CiMgVG90YWwgcG9wdWxhdGlvbgpnZ3Bsb3Qoc3JnICU+JSBmaWx0ZXIoIWlzLm5hKHNleC5yYXRpbykpICAsIGFlcyh4ID0geWVhciwgeSA9IEZlbWFsZSArIE1hbGUpKSArCiAgICAgICAgIGdlb21fcG9pbnQoKSArIAogICAgICAgICBnZW9tX2xpbmUoKSArCiAgICBzY2FsZV94X2NvbnRpbnVvdXMoYnJlYWtzID0gc2VxKG1pbihzcmckeWVhciksIG1heChzcmckeWVhciksIDUpLCBtaW5vcl9icmVha3MgPSBjKG1pbihzcmckeWVhcik6IG1heChzcmckeWVhcikpKSArIAogIHNjYWxlX3lfY29udGludW91cyhicmVha3MgPSBzZXEoMCwxLDAuMikpICsKICBsYWJzKHkgPSAnTnVtYmVyIG9mIGFkdWx0IGtha2FwbycpIAogIAoKIyBNYWxlIGFuZCBmZW1hbGUgcG9wdWxhdGlvbgpnZ3Bsb3Qoc3JnICU+JSBmaWx0ZXIoIWlzLm5hKHNleC5yYXRpbykgJiAhaXMubmFuKHByb3AuY2x1dGNoKSkgJT4lIHBpdm90X2xvbmdlcihjb2xzID0gRmVtYWxlOk1hbGUsIG5hbWVzX3RvID0gJ3ZhcmlhYmxlJykgLCBhZXMoeCA9IHllYXIsIHkgPSB2YWx1ZSwgY29sb3VyID0gdmFyaWFibGUpKSArCiAgICAgICAgIGdlb21fcG9pbnQoKSArIAogICAgICAgICBnZW9tX2xpbmUoKSArCiAgZmFjZXRfd3JhcCh+dmFyaWFibGUsIHNjYWxlcyA9ICdmaXhlZCcpICsgCiAgZ3VpZGVzKGNvbG91ciA9ICdub25lJykgKyAKICAgIHNjYWxlX3hfY29udGludW91cyhicmVha3MgPSBzZXEobWluKHNyZyR5ZWFyKSwgbWF4KHNyZyR5ZWFyKSwgNSksIG1pbm9yX2JyZWFrcyA9IGMobWluKHNyZyR5ZWFyKTogbWF4KHNyZyR5ZWFyKSkpICsgCiAgbGFicyh5ID0gJ051bWJlciBvZiBhZHVsdCBrYWthcG8nKQogIAojIFNleCByYXRpbwpnZ3Bsb3Qoc3JnICU+JSBmaWx0ZXIoIWlzLm5hKHNleC5yYXRpbykmICFpcy5uYW4ocHJvcC5jbHV0Y2gpKSAsIGFlcyh4ID0geWVhciwgeSA9IHNleC5yYXRpbykpICsKICAgICAgICAgZ2VvbV9wb2ludCgpICsgCiAgICAgICAgIGdlb21fbGluZSgpICsKICAgIHNjYWxlX3hfY29udGludW91cyhicmVha3MgPSBzZXEobWluKHNyZyR5ZWFyKSwgbWF4KHNyZyR5ZWFyKSwgNSksIG1pbm9yX2JyZWFrcyA9IGMobWluKHNyZyR5ZWFyKTogbWF4KHNyZyR5ZWFyKSkpICsgCiAgbGFicyh5ID0gJ0Y6TSBhZHVsdCBzZXggcmF0aW8nKQoKYGBgCkNoYW5nZSBpbiBtdWx0aXBsZSBjb3B1bGF0aW9uIHJhdGUgb3ZlciB0aW1lOgoKYGBge3IgdGltZV9tdWx0X2NvcHVsYXRpb259CiMgU2V4IHJhdGlvCmdncGxvdChzcmcgJT4lIGZpbHRlcighaXMubmEoc2V4LnJhdGlvKSYgIWlzLm5hbihwcm9wLmNsdXRjaCkpICwgYWVzKHggPSB5ZWFyLCB5ID0gcHJvcC5jbHV0Y2gpKSArCiAgICAgICAgIGdlb21fcG9pbnQoKSArIAogICAgICAgICBnZW9tX2xpbmUoKSArCiAgc2NhbGVfeF9jb250aW51b3VzKGJyZWFrcyA9IHNlcShtaW4oc3JnJHllYXIpLCBtYXgoc3JnJHllYXIpLCA1KSwgbWlub3JfYnJlYWtzID0gYyhtaW4oc3JnJHllYXIpOiBtYXgoc3JnJHllYXIpKSkgKyAKICBzY2FsZV95X2NvbnRpbnVvdXMoYnJlYWtzID0gc2VxKDAsMSwwLjIpKSArCiAgbGFicyh5ID0gJ1Byb3BvcnRpb24gb2YgbXVsdGlwbGUgY29wdWxhdGlvbnMnKQoKYGBgCgojIFNlc3Npb24gaW5mb3JtYXRpb24KCmBgYHtyIHNlc3Npb259CnNlc3Npb25JbmZvKCkKYGBgCg==
