# Supplementary figures and images for: Hidden impacts of conservation management on fertility of the critically endangered kākāpō
Source: PeerJ. 2023 Feb 3;11:e14675. doi: 10.7717/peerj.14675 (PMC9901309; doi:10.7717/peerj.14675)

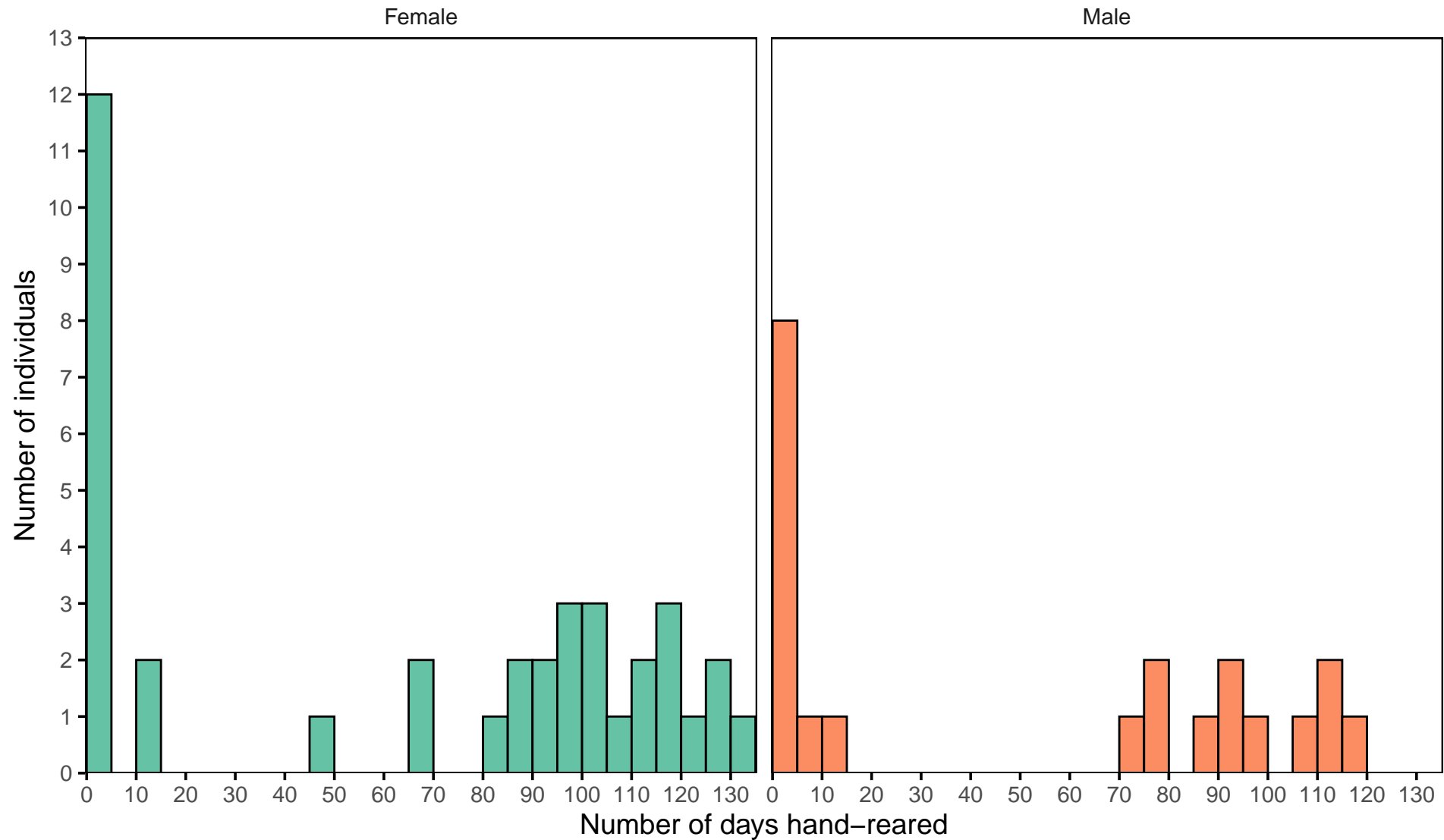

Supplement: Supplemental Information 2 — Distribution of the number of days for which kākāpō were hand-reared, from the 59 kākāpō (38 females and 21 males) hatched after intensive management and hand-rearing began which contributed to the data set of 225 clutches [file peerj-11-14675-s002.pdf]
