# Supplementary material for: Hidden impacts of conservation management on fertility of the critically endangered kākāpō
Source: PeerJ. 2023 Feb 3;11:e14675. doi: 10.7717/peerj.14675 (PMC9901309; doi:10.7717/peerj.14675)
Supplement: Supplemental Information 3 — Distributions for the numeric fixed parameters in the Bayesian model of clutch fertility. [file peerj-11-14675-s003.pdf]

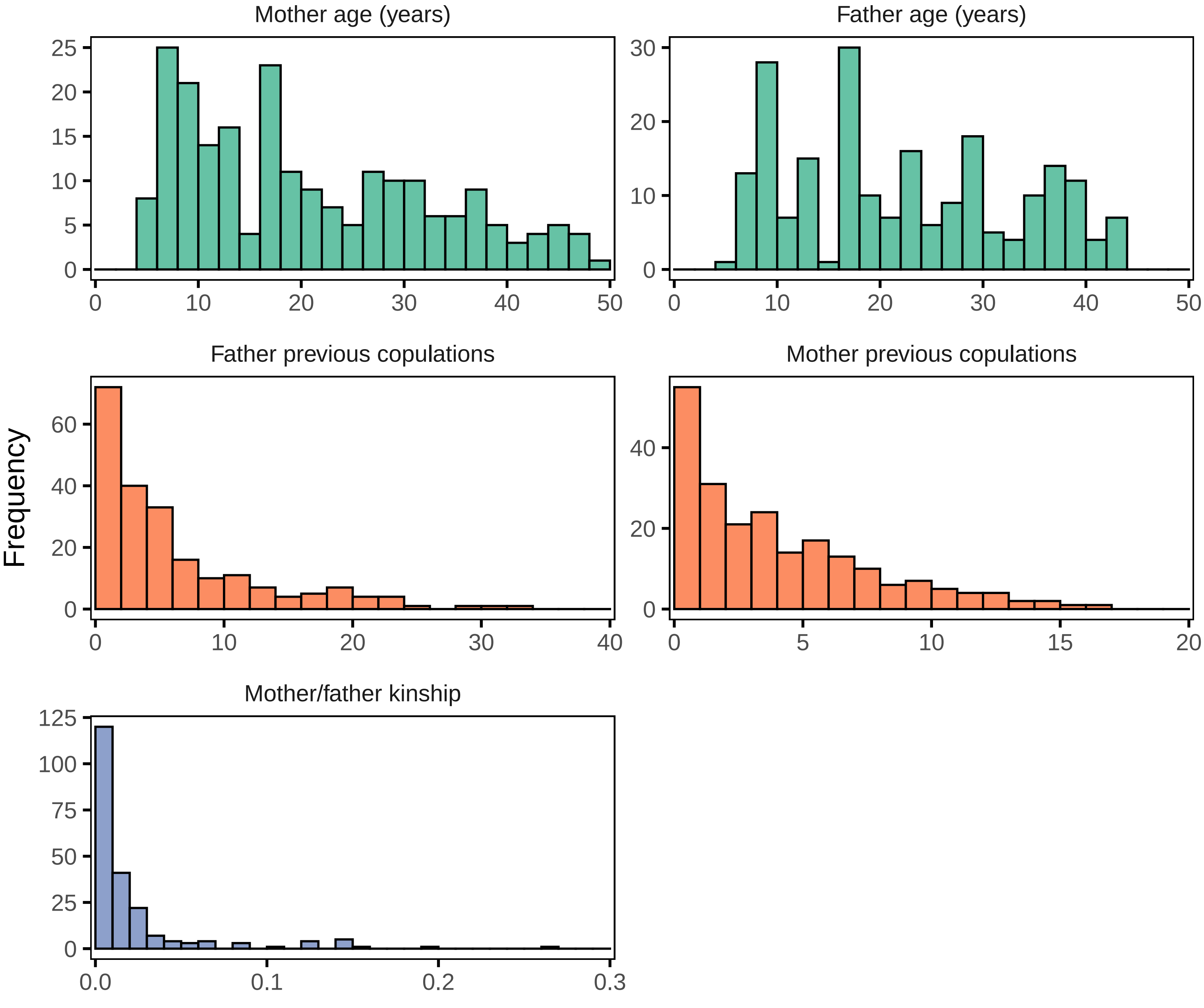
Mother age (years)
Father age (years)
Father previous copulations
Mother previous copulations
Mother/father kinship
Frequency
0
5
10
15
20
25
30
40
50
60
75
100
125
0.0
0.1
0.2
0.3
