# Supplementary material for: Hidden impacts of conservation management on fertility of the critically endangered kākāpō
Source: PeerJ. 2023 Feb 3;11:e14675. doi: 10.7717/peerj.14675 (PMC9901309; doi:10.7717/peerj.14675)
Supplement: Supplemental Information 4 — The likelihood of paternity, given the order in which males copulated with the female. These probabilities are calculated from clutches produced by the female copulating with multiple males which had genetically confirmed paternity. [file peerj-11-14675-s004.pdf]

Mating order	Probability of paternity
First	0.41
First & last	0.80
Last	0.59
Middle	0.17
